# Molecular and Clinical Characterization of CD80 Expression *via* Large-Scale Analysis in Breast Cancer

**DOI:** 10.3389/fphar.2022.869877

**Published:** 2022-06-22

**Authors:** Qin Zhang, Chaowei Gao, Jianqiang Shao, Shengze Zhang, Peng Wang, Zunyi Wang

**Affiliations:** ^1^ Thyroid and Breast Department III, Cangzhou Central Hospital, Cangzhou, China; ^2^ Breast Surgery Department, Chongqing University Three Gorges Hospital, Chongqing, China

**Keywords:** breast cancer, CD80, immune response, inflammatory activity, microenvironment

## Abstract

Cancer immunotherapy is emerging as a novel promising therapy option for cancer patients. Despite the critical role of CD80 in the regulation of immune responses, the expression and biological functions of CD80 in breast cancer remain unknown. In this study, we aimed to investigate the role of CD80 both clinically and molecularly in breast cancer at a transcriptome level. Herein, we first analyzed the transcriptome profile and relevant clinical information derived from a total of 1090 breast cancer patients recorded in The Cancer Genome Atlas database and then validated this in the Molecular Taxonomy of Breast Cancer International Consortium (METABRIC) database (*n* = 1904). We revealed the associations of CD80 and the main molecular and clinical characteristics of breast cancer. The gene ontology analysis and Gene Set Variation Analysis of the CD80-related genes revealed that CD80 was closely correlated with immune responses and inflammatory activities in breast cancer. Moreover, the CD80 expression showed a remarkable positive correlation with several infiltrated immune cell populations. In summary, the CD80 expression was closely correlated with the malignancy of breast cancer, and our findings suggest that CD80 might be a promising target for immunotherapeutic strategies. To the best of our knowledge, this is the first integrative study characterizing the role of the CD80 expression in breast cancer *via* large-scale analyses.

## Introduction

Breast cancer represents the world’s most prevalent cancer and has now surpassed lung cancer as the leading cause of death globally (Miller et al., 2020). Escaping tumor cells from destruction induced by the immune system represents an important hallmark of cancer (Hanahan and Weinberg, 2011). One of the mechanisms of immune escape is by inducing an exhausted phenotype in effector lymphocytes and further preventing effective antitumor effects (Barber et al., 2006). It has been well established that complex interactions of the receptor and ligand are involved in regulating T-cell activation and inducing checkpoint control of T-cell effector functions (Chauvin and Zarour, 2020). More recently, cancer immunotherapy has been emerging as a novel promising therapy option for cancer patients. Owing to the evident prognosis benefits of cancer immunotherapy, antibodies targeting the negative immune checkpoint molecules programmed cell death protein 1 (PD-1) or PD-1 ligand 1 (PD-L1), and cytotoxic T-lymphocyte-associated protein 4 (CTLA-4) (Hodi et al., 2010) has been approved by the FDA in the treatment of several cancer entities. However, only part of the patients responded to the treatment by blocking the immune checkpoint axis (Brahmer et al., 2012; Topalian et al., 2012; Bersanelli and Buti, 2017). This phenomenon has raised our interest in investigating other potential molecules involved in these immune response regulations.

CD80 is a member of the B7 family and the immunoglobulin superfamily, which is composed of molecules present in antigen-presenting cells (APCs) and their receptors present on the T cells (Mir, 2015). Previous studies have showed that CD80 is the ligand for the proteins CD28 involved in autoregulation and intercellular association and CTLA-4 (an important immune checkpoint molecule) expressed on the surface of T cells (Peach et al., 1995; van der Merwe et al., 1997; Mir, 2015; Chen et al., 2020). Interactions of CD80, CD28, and CTLA-4 played a complicated role in the immunological synapse in T- and B-cell activation, proliferation, and differentiation (Bhatia et al., 2005). The interaction between CD80 and CD28, together with TCR and MHC interaction, induces the activation of nuclear factor‐κB (NF-κB), mitogen‐activated protein kinase (MAPK), and the calcium–calcineurin pathway, thereby playing a diverse role in manipulating both the innate and the adaptive immune system (van der Merwe et al., 1997; Zheng et al., 2004; Chen et al., 2020). Given the role of CD80 in regulating the immune system is complicated, providing the opportunity for CD80 interactions to be involved in various diseases including multiple autoimmune diseases (Windhagen et al., 1995; Wong et al., 2005; Nolan et al., 2008), and various cancers (Yang et al., 2006; Imade et al., 2013). Among cancers, previous studies have reported that low surface expression of CD80 was associated with the immune escape mechanism of colon cancer, and upregulating CD80 on tumor cell surface successfully enhances antitumor immune responses (Bhatia et al., 2006). Despite multiple studies supporting that CD80 plays a critical role in regulating adaptive and innate immunity during tumor progression, the role of CD80 and its association with the tumor immune microenvironment in breast cancer remains largely unknown.

Taking advantage of the TCGA database, a comprehensive analysis of a large-scale CD80-related transcriptome profile was carried out, which revealed the potential role of CD80 in immune response and inflammatory activities. Furthermore, our findings were well-validated in another RNA-seq dataset of 1994 samples obtained from the METABRIC database. To the best of our knowledge, this is the first and largest study investigating the landscape of CD80 expression in breast cancer both molecularly and clinically.

## Materials and Methods

### Sample and Data Collection

We followed the methods of our previous study (Zhang et al., 2021)*.* The RNA-sequencing data of TCGA were downloaded and analyzed using GDCRNATools (Li et al., 2018) in R language. Raw count data were normalized using the TMM method implemented in edgeR (Robinson et al., 2010) and were then transformed by the voom method in the limma (Ritchie et al., 2015) package; only genes with cpm >1 in more than half of the samples were selected for further analyses. Standardized survival information from the TCGA database was retrieved from the TCGA Pan-Cancer Clinical Data Resource (TCGA-CDR) (Liu et al., 2018a). The METABRIC database (Curtis et al., 2012) including transcriptome and clinical information data of a total of 1904 breast cancer cases were retrieved from the cBioPortal database.

### Bioinformatics Analysis and Statistical Analysis

Gene enrichment analyses of the genes closely related to CD80 were performed using the clusterProfiler package (Yu et al., 2012) inR. Immune-related genes were collected from the Immunology Database and Analysis Portal (ImmPort) database (Bhattacharya et al., 2014). The Microenvironment Cell Populations-counter algorithm (Becht et al., 2016) was applied to estimate the absolute abundance of immune cell populations of the tumor. The GSVA analysis (Hänzelmann et al., 2013) was carried out to estimate the scores of metagenes that are related to immune functions and inflammatory activities (Rody et al., 2009). The Pearson correlation method was applied to estimate the correlations between continuous variables; genes with at least moderate correlation with CD80 were defined as |R|>0.4 and *p* < 0.05. R denotes the correlation coefficient. The greater the absolute value of the correlation coefficient is, the stronger the correlation is: the closer the correlation coefficient is to 1 or −1, the stronger the correlation is; the closer the correlation coefficient is to 0, the weaker the correlation is. Potential differences in variables between groups were determined using the Student t-test, one-way ANOVA, or Pearson’s chi-squared test. All statistical tests and graphical work were carried out through R software (version 3.6.4) and associated packages including circlize (Gu et al., 2014), pheatmap, ggplot2 (Hadley, 2011), and corrgram (Friendly, 2002). All statistical tests were two-sided. The *p*-value of less than 0.05 was considered statistically significant.

## Results

### Expression Pattern of CD80 among Multiple Cancer Sites

**FIGURE 1:**
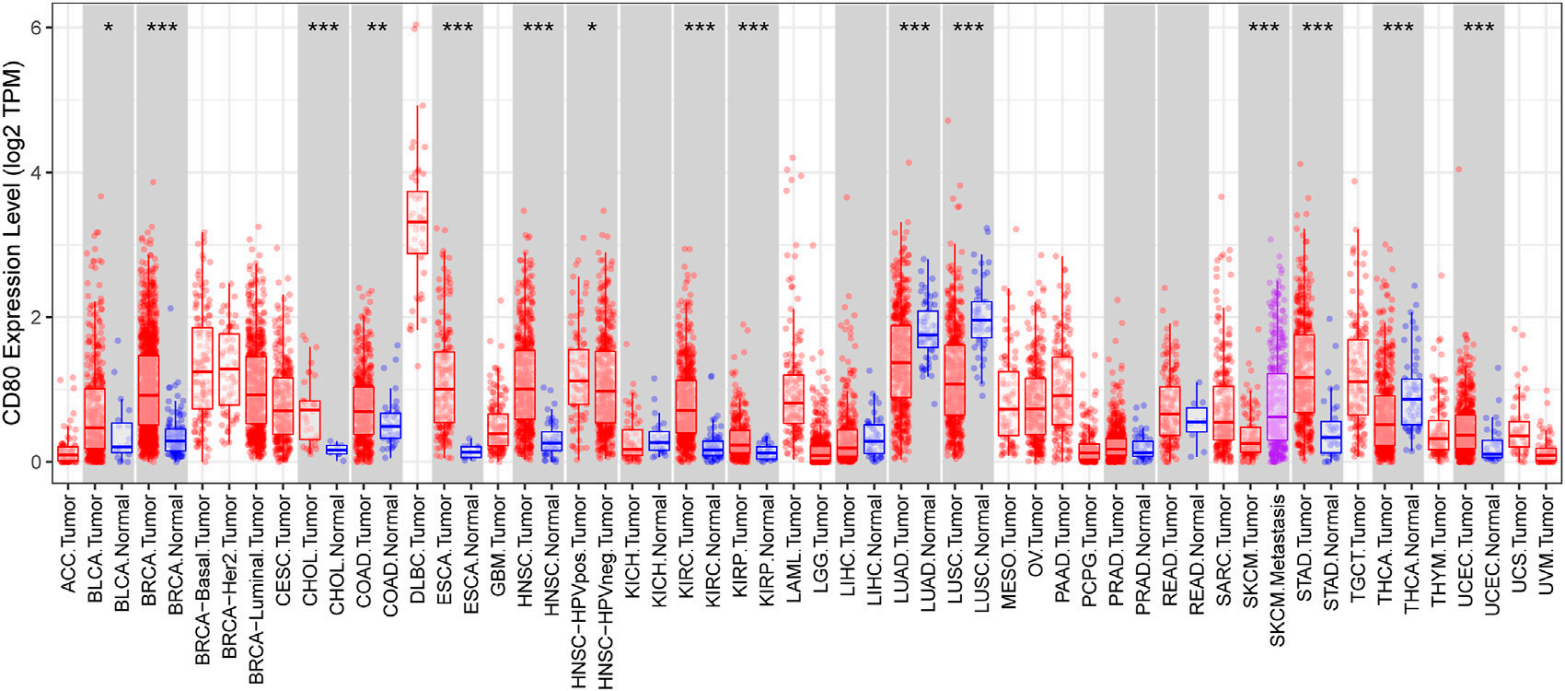
CD80 expression status in pan-cancer. CD80 expression levels in all tumors and adjacent normal tissues across TCGA (*p < 0.05, **p < 0.01, and ***p < 0.001).

**FIGURE 2:**
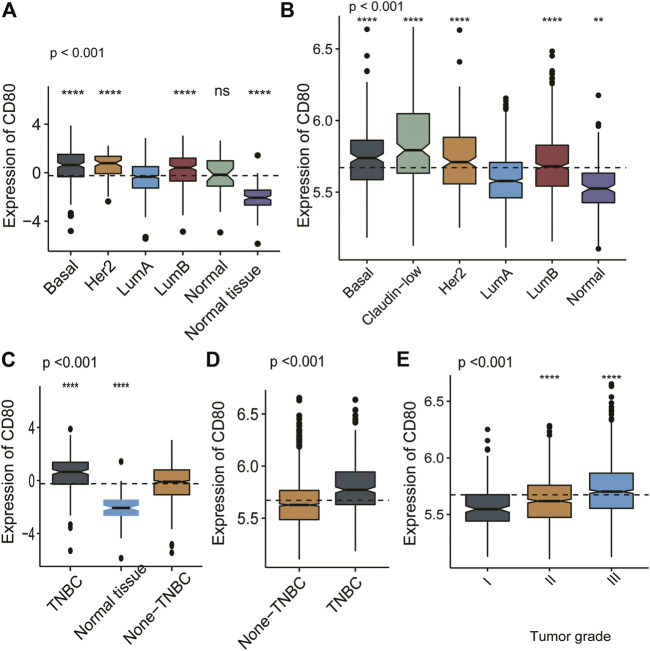
CD80 expression in different molecular subtypes of the transcriptional classification scheme in TCGA and METABRIC cohort. Expression pattern of CD80 in TCGA database **(A,C)**, and in METABRIC database **(B,D,E)**. (*p < 0.05, **p < 0.01, ***p < 0.001, and ***p < 0.0001).

We depicted the expression pattern of CD80 among multiple cancer sites (Figure 1). Intriguingly, we found CD80 showed significantly higher expression in several cancers including BLCA (bladder urothelial carcinoma), BRCA (breast invasive carcinoma), CHOL (Cholangiocarcinoma), COAD (colon adenocarcinoma), ESCA (esophageal carcinoma), HNSC (head and neck squamous cell carcinoma), KIRC (kidney renal clear cell carcinoma), KIRP (kidney renal papillary cell carcinoma), STAD (stomach adenocarcinoma), and UCEC (uterine corpus endometrial carcinoma). To explore the expression pattern of CD80 in breast cancer, we further analyzed the large-scale transcriptome data on breast cancer from TCGA and METABRIC databases. We listed the association between CD80 and clinical characteristics of breast cancer in Tables 1, 2. CD80 expression was elevated in basal-like and HER2-enriched subtype when compared with the luminal A subtype in TCGA database (*n* = 1090) (Figure 2A), and we also observed this result in the METABRIC database (*n* = 1904) (Figure 2B). Furthermore, we found CD80 showed higher expression in triple-negative breast cancer (TNBC) when compared with the non-TNBC group (Figures 2C,D). In addition, we also observed elevated expression of CD80 in higher tumor grades (Figure 2E). Furthermore, The Human Protein Atlas was used in our study to evaluate the immunohistochemistry (IHC) data pertaining to the protein expression of CD80 in breast cancer and normal tissue (Supplementary Figure S1). In summary, our results showed that CD80 expression was associated with the malignancy of breast cancer.

**TABLE 1:** Association between the CD80 mRNA expression and clinicopathologic characteristics in TCGA cohort.

	Expression			
	Total (*n* = 1090)	CD80 high (*n* = 545)	CD80 low (*n* = 545)	*p*-value
Age (years)				
≥55	517 (47.4%)	264 (48.4%)	253 (46.4%)	0.544
<55	573 (52.6%)	281 (51.6%)	292 (53.6%)	
T stage				
T1	279 (25.6%)	139 (25.5%)	140 (25.7%)	0.0285
T2	631 (57.9%)	333 (61.1%)	298 (54.7%)	
T3	137 (12.6%)	53 (9.7%)	84 (15.4%)	
T4	40 (3.7%)	19 (3.5%)	21 (3.9%)	
Unknown	3 (0.3%)	1 (0.2%)	2 (0.4%)	
N stage				
N0	514 (47.2%)	267 (49.0%)	247 (45.3%)	0.195
N1	360 (33.0%)	166 (30.5%)	194 (35.6%)	
N2	120 (11.0%)	67 (12.3%)	53 (9.7%)	
N3	76 (7.0%)	40 (7.3%)	36 (6.6%)	
Unknown	20 (1.8%)	5 (0.9%)	15 (2.8%)	
M stage				
M0	907 (83.2%)	462 (84.8%)	445 (81.7%)	0.351
M1	22 (2.0%)	9 (1.7%)	13 (2.4%)	
Unknown	161 (14.8%)	74 (13.6%)	87 (16.0%)	
AJCC stage				
I	181 (16.6%)	85 (15.6%)	96 (17.6%)	0.587
II	621 (57.0%)	319 (58.5%)	302 (55.4%)	
III	250 (22.9%)	125 (22.9%)	125 (22.9%)	
IV	20 (1.8%)	8 (1.5%)	12 (2.2%)	
Unknown	18 (1.7%)	8 (1.5%)	10 (1.8%)	
ER status				
Negative	236 (21.7%)	162 (29.7%)	74 (13.6%)	<0.001
Positive	803 (73.7%)	359 (65.9%)	444 (81.5%)	
Unknown	51 (4.7%)	24 (4.4%)	27 (5.0%)	
PR status				
Negative	343 (31.5%)	205 (37.6%)	138 (25.3%)	<0.001
Positive	694 (63.7%)	315 (57.8%)	379 (69.5%)	
Unknown	53 (4.9%)	25 (4.6%)	28 (5.1%)	
HER2 status				
Negative	895 (82.1%)	435 (79.8%)	460 (84.4%)	0.0135
Positive	168 (15.4%)	100 (18.3%)	68 (12.5%)	
Unknown	27 (2.5%)	10 (1.8%)	17 (3.1%)	

**TABLE 2:** Association between the CD80 mRNA expression and clinicopathologic characteristics in the METABRIC cohort.

	Expression			
	Total (*n* = 1904)	CD80 high (*n* = 952)	CD80 low (*n* = 952)	*p*-value
Age (years)				
≥55	952 (50.0%)	488 (51.3%)	464 (48.7%)	0.292
<55	952 (50.0%)	464 (48.7%)	488 (51.3%)	
Tumor size				
≥2 cm	592 (31.1%)	273 (28.7%)	319 (33.5%)	0.0277
<2 cm	1292 (67.9%)	668 (70.2%)	624 (65.5%)	
Unknown	20 (1.1%)	11 (1.2%)	9 (0.9%)	
AJCC stage				
0	4 (0.2%)	2 (0.2%)	2 (0.2%)	0.169
I	475 (24.9%)	223 (23.4%)	252 (26.5%)	
II	800 (42.0%)	431 (45.3%)	369 (38.8%)	
III	115 (6.0%)	63 (6.6%)	52 (5.5%)	
IV	9 (0.5%)	4 (0.4%)	5 (0.5%)	
Unknown	501 (26.3%)	229 (24.1%)	272 (28.6%)	
Tumor Grade				
I	165 (8.7%)	47 (4.9%)	118 (12.4%)	<0.001
II	740 (38.9%)	328 (34.5%)	412 (43.3%)	
III	927 (48.7%)	548 (57.6%)	379 (39.8%)	
Unknown	72 (3.8%)	29 (3.0%)	43 (4.5%)	
ER status				
Negative	445 (23.4%)	300 (31.5%)	145 (15.2%)	<0.001
Positive	1459 (76.6%)	652 (68.5%)	807 (84.8%)	
PR status				
Negative	895 (47.0%)	523 (54.9%)	372 (39.1%)	<0.001
Positive	1009 (53.0%)	429 (45.1%)	580 (60.9%)	
HER2 status				
Negative	1668 (87.6%)	811 (85.2%)	857 (90.0%)	0.00175
Positive	236 (12.4%)	141 (14.8%)	95 (10.0%)	

### CD80 Was Closely Related to Immune Functions in Breast Cancer

**FIGURE 3:**
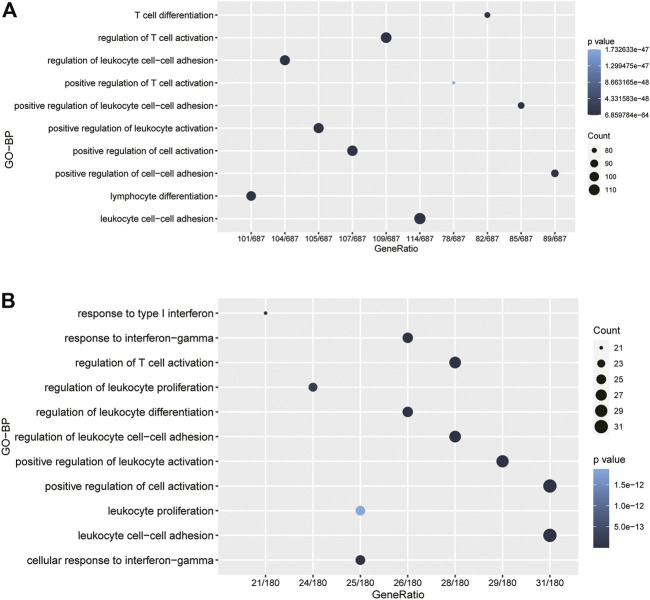
CD80 was closely related to immune functions in breast cancer. Gene ontology analysis showed that CD80 was mainly involved in immune response and inflammatory response in the TCGA and METABRIC cohorts **(A,B)**.

To further investigate the potential biological role of CD80, we screened out gene sets correlated with CD80 expression using the TCGA and METABRIC databases, respectively; these results are provided in Supplementary Tables S1, S2. Then, functional enrichment analyses were performed with these two gene sets using the clusterProfiler algorithm in R (Yu et al., 2012). Interestingly, we found CD80-related genes were mainly enriched in inflammatory and immune-related biological processes, when these biological processes were sorted by *p*-value in an increasing order, including biological processes correlated with the regulation of T-cell activation, regulation of leukocyte activation, and regulation of leukocyte cell−cell adhesion (Figures 3A,B). Generally, these results were mutually validated in TCGA and METABRIC databases.

### CD80-Related Immune Response

**FIGURE 4:**
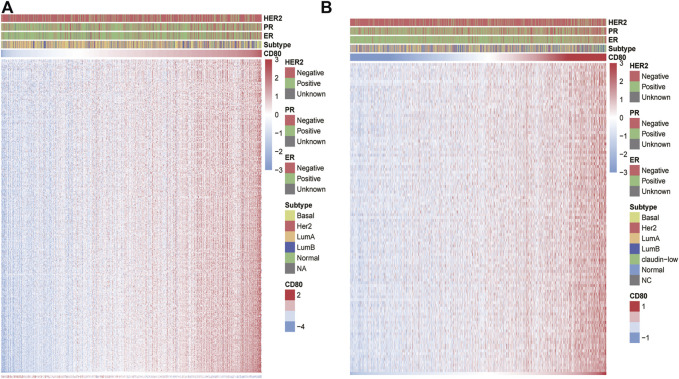
CD80-related immune responses. Most immune-related genes are positively correlated with the CD80 expression in the TCGA and METABRIC databases, while few genes are negatively associated **(A,B)**.

To further investigate the potential biological functions of CD80 in immune response in breast cancer, we retrieved a total of 4723 immune-related genes from The Immunology Database and Analysis Portal (ImmPort) database (https://www.immport.org/shared/home). To characterize the correlation pattern of CD80 and immune-related genes, we screened the genes that were most relevant to CD80, with a cut-off value of |R|>0.4 and *p* < 0.05. Interestingly, we found a total of 394 and 110 genes were positively correlated with CD80 expression in TCGA and METABRIC databases (Supplementary Table S3), respectively, while only 3 and 0 genes were negatively correlated with CD80 expression, respectively (Figures 4A,B). Our results indicated that CD80 was positively correlated with the most relevant immune responses and negatively correlated with few immune responses in breast cancer.

### Association of CD80 Expression and Infiltrated Cells in the Tumor Microenvironment

**FIGURE 5:**
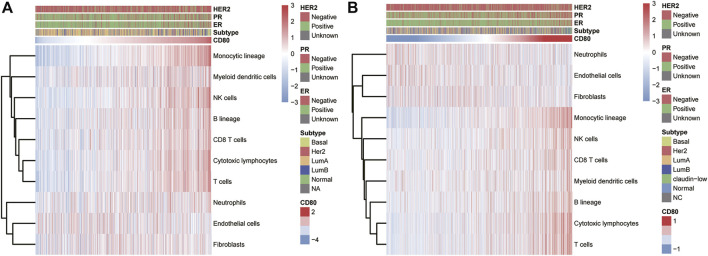
Association between the CD80 expression and immune cell populations in the TCGA and METABRIC cohort **(A,B)**.

To further reveal the functional role of CD80 in the breast cancer immune microenvironment, we estimated the absolute abundance of eight immune and two stromal cell populations from the transcriptome data through the Microenvironment Cell Populations-counter method developed by Etienne Becht et al. (2016). We found CD80 expression was positively correlated with monocytic lineage, myeloid dendritic cells, T cells, NK cells, B lineage, CD8 T cells, and cytotoxic lymphocytes but not with endothelial cells, fibroblasts, and neutrophils (Figure 5). Interestingly, these results can be mutually validated well in both TCGA and METABRIC databases.

### CD80 Expression Was Relevant to Inflammatory Activities

**FIGURE 6:**
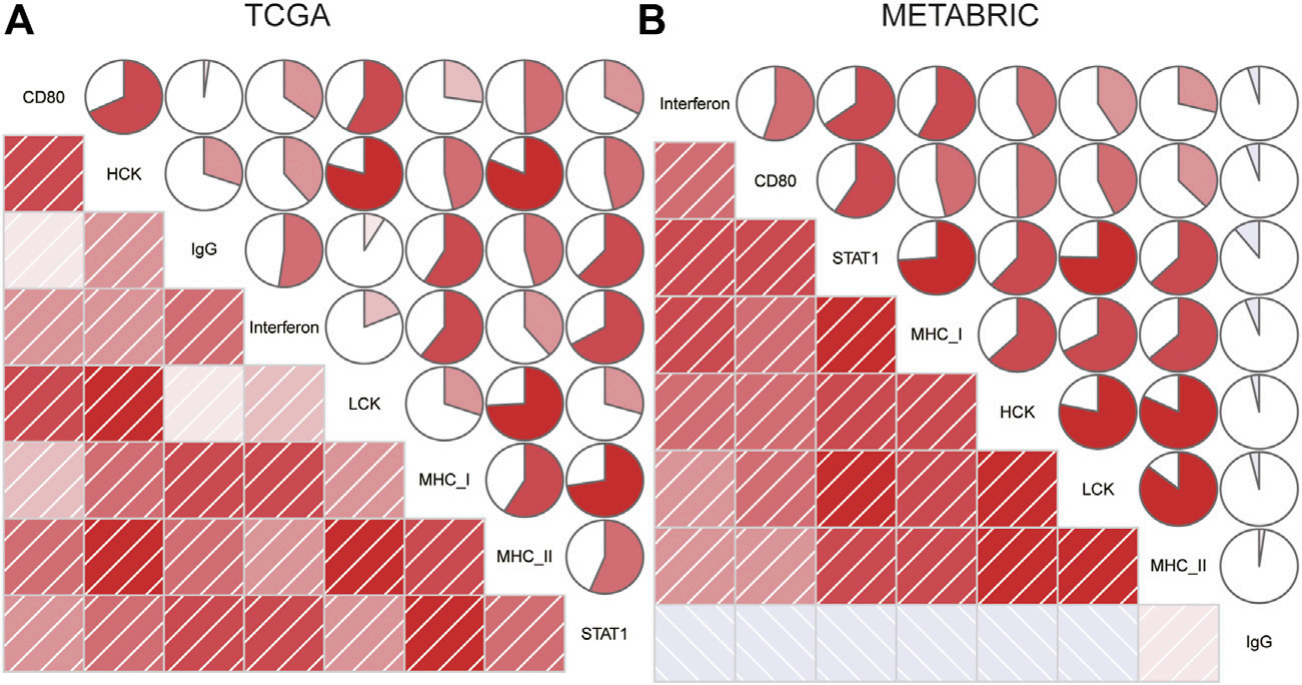
Relationship between the CD80 expression and inflammatory activities in the TCGA and METABRIC cohort **(A,B)**.

To further clarify the role of CD80-related inflammatory activities, we subsequently defined seven clusters of metagenes based on 104 genes (Liu et al., 2018b) (Supplementary Table S3), indicating different types of immune response and inflammation. Interestingly, we observed that CD80 expression was positively correlated with HCK, interferon, LCK, MHC-I, MHC-II, and STAT1 in TCGA database (Figure 6A), and this result can be well-validated in the METABRIC database (Figure 6B). The aforementioned results showed that CD80 was involved in the T-cell signaling transduction, activation of macrophages, and antigen-presenting cells. However, no strong association between CD80 expression and IgG was found. In summary, these results further confirmed the important role of CD80 in the breast cancer immune microenvironment.

## Discussion

Breast cancer is the leading cause of deaths and affects human health severely, reinforcing the urgent need for new therapeutic options. Most recently, cancer immunotherapy has been an emerging promising treatment option for patients with TNBC. In the past few years, most studies regarding the field of cancer immunotherapy have been focusing on a few checkpoint molecules including, PD-1/PD-L1 and CTLA-4. Owing to the complex interactions and roles of tumor immune modulators, previous strategies might be insufficient to inhibit tumor progression.

In the past few decades, most of the studies have been focused on the role of PD1/PDL1 and CTLA-4 in breast cancer (Hodi et al., 2010), while few studies paid attention to the potential role of CD80. A previous study reported that the CD80 expression was higher in MDA-MB-468, MCF-7, and MDA-MB-231 breast cancer cells than in normal MCF10A cells (Li et al., 2020). Evidence supported that pharmacological activation of TP53 can promote the CD80 expression in human cancer cells originating from the epithelium (Scarpa et al., 2021). Previous studies have showed that the CD80 expression is lower in several cancer cells, and the loss of CD80 alone promotes their ability to escape the attack from the immune system and imparts energy and apoptosis in tumor-infiltrating T cells (Tirapu et al., 2006). CD80 has also been reported to promote the memory response in cytotoxic T lymphocytes (CTLs) (Wang et al., 2013), which suggests that the CD80 expression on the tumor is involved in antitumor CTL effector function. In the present study, we analyzed the CD80 expression pattern in breast cancer *via* a total of 2994 breast cancer samples. We found the CD80 expression was associated with the higher malignant pathological type of breast cancer. Interestingly, our results showed that CD80 was significantly downregulated in several cancer types including LUAD (lung adenocarcinoma), LUSC (lung squamous cell carcinoma), and THCA (thyroid cancer); these results suggest that the role of CD80 might be varied by cancer types. Moreover, we observed that the association between the CD80 expression and immune and inflammatory responses is similar to the pattern of PD1 in breast cancer (Liu et al., 2020). In summary, our results suggest that CD80 and PD1 might play a synergistic role in regulating immune and inflammatory responses to promote tumor progression.

Taken together, the CD80 expression was closely correlated with tumor malignancy in breast cancer. Notably, CD80 might play important roles in regulating not only T-cell immune functions but also other immune cells, thereby regulating antitumor immune effects, Moreover, the strong correlation between CD80 and other immune genes suggests the possibility of co-regulating the immune microenvironment in breast cancer, which provides novel insights for targeting the combination of immune checkpoint members and CD80. To the best of our knowledge, this is the first integrative study characterizing the molecular and clinical features of CD80 in breast cancer *via* large-scale molecular data. Our findings further suggest that CD80 might be a promising target for immunotherapy in breast cancer; future studies are warranted to elaborate on the potential co-regulatory role of CD80 and other immune checkpoint members.

## Data Availability

The datasets presented in this study can be found in online repositories. The names of the repository/repositories and accession number(s) can be found in the article/Supplementary Material.
