# Supplementary figures and images for: Molecular and Clinical Characterization of CD80 Expression via Large-Scale Analysis in Breast Cancer
Source: Front Pharmacol. 2022 Jun 22;13:869877. doi: 10.3389/fphar.2022.869877 (PMC9257272; doi:10.3389/fphar.2022.869877)

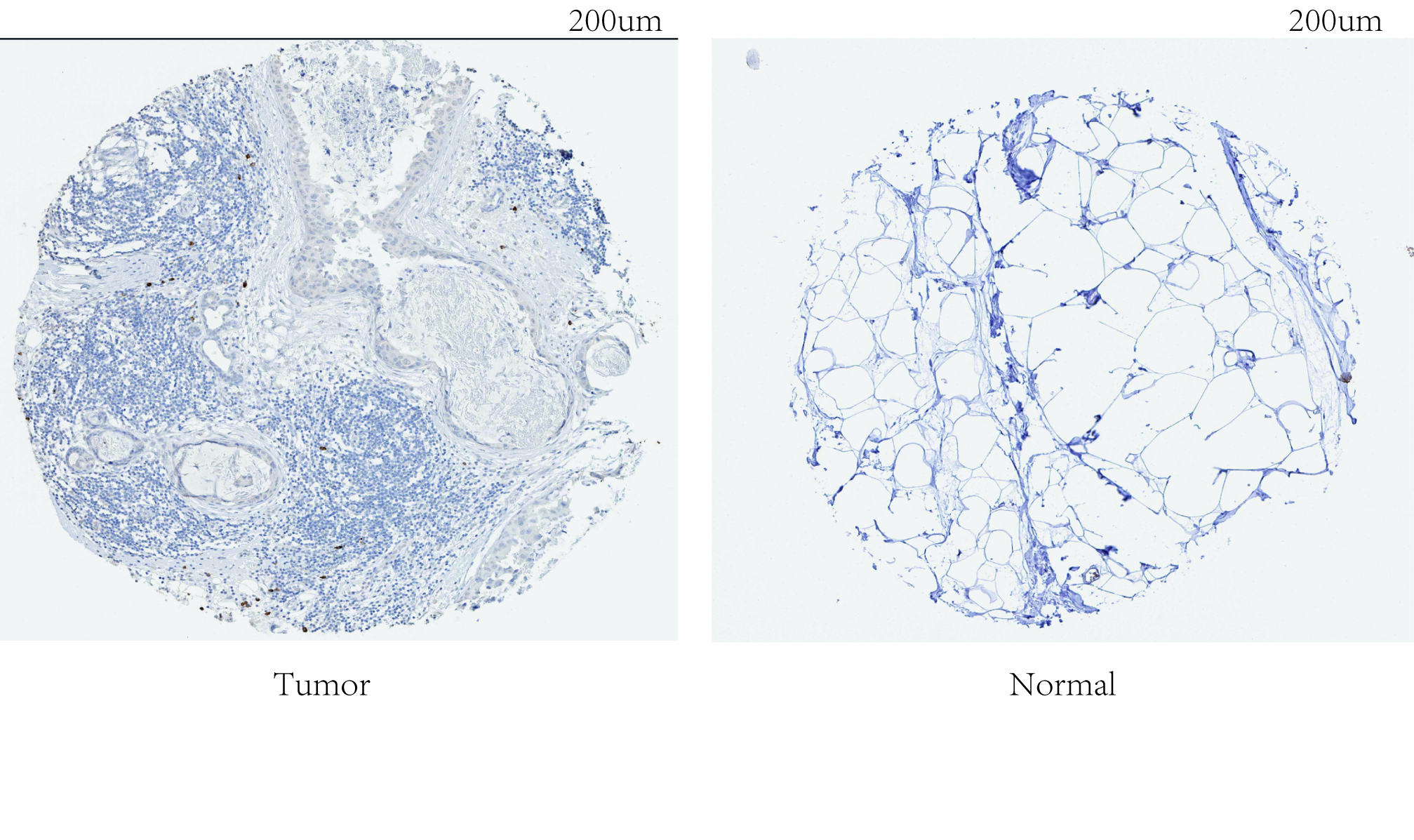

Supplement: Supplementary file 1 [file Image1.tif]
