# Supplementary material for: Molecular and Clinical Characterization of CD80 Expression via Large-Scale Analysis in Breast Cancer
Source: Front Pharmacol. 2022 Jun 22;13:869877. doi: 10.3389/fphar.2022.869877 (PMC9257272; doi:10.3389/fphar.2022.869877)
Supplement: Supplementary file 2 [file DataSheet1.docx]

**Supplementary File lists**

**Table S1. Genes correlated with CD80 in TCGA cohort.** Genes with Pearson |R| ≥ 0.4. (pvalue=0 denotes p<0.001)

**Table S2. Genes correlated with CD80 in METABRIC cohort.** Genes with Pearson |R| ≥ 0.4.

**Table S3 The immune-related positively correlated genes lists.** Genes with Pearson R≥ 0.4.

**Table S4 Seven clusters of 104 genes defined as metagenes.**

**Table S1 Genes correlated with CD80 in TCGA cohort**

|  | | | | | | | |
| --- | --- | --- | --- | --- | --- | --- | --- |
|  | | symbol | | correlation | | pvalue | |
| 1 | | CD80 | | 1 | | 0 | |
| 2 | | CD86 | | 0.817542 | | 0 | |
| 3 | | EPSTI1 | | 0.815768 | | 0 | |
| 4 | | TFEC | | 0.79939 | | 0 | |
| 5 | | PIK3AP1 | | 0.796826 | | 0 | |
| 6 | | CCR1 | | 0.793837 | | 0 | |
| 7 | | FPR3 | | 0.791827 | | 0 | |
| 8 | | TNFSF13B | | 0.778435 | | 0 | |
| 9 | | HAVCR2 | | 0.770855 | | 0 | |
| 10 | | SLC7A7 | | 0.766504 | | 0 | |
| 11 | | LILRB1 | | 0.751076 | | 0 | |
| 12 | | PTPRO | | 0.750374 | | 0 | |
| 13 | | SIGLEC1 | | 0.750103 | | 0 | |
| 14 | | GBP5 | | 0.749994 | | 0 | |
| 15 | | LCP2 | | 0.743427 | | 0 | |
| 16 | | PDCD1LG2 | | 0.743223 | | 0 | |
| 17 | | PLEK | | 0.742064 | | 0 | |
| 18 | | HPSE | | 0.741456 | | 0 | |
| 19 | | FCGR3A | | 0.740713 | | 0 | |
| 20 | | CLEC7A | | 0.740121 | | 0 | |
| 21 | | CXCL10 | | 0.74004 | | 0 | |
| 22 | | CYBB | | 0.730124 | | 0 | |
| 23 | | SAMSN1 | | 0.726823 | | 0 | |
| 24 | | LILRB4 | | 0.725727 | | 0 | |
| 25 | | LILRB2 | | 0.72467 | | 0 | |
| 26 | | CD84 | | 0.722943 | | 0 | |
| 27 | | CD53 | | 0.722942 | | 0 | |
| 28 | | RSAD2 | | 0.721358 | | 0 | |
| 29 | | SP140 | | 0.720884 | | 0 | |
| 30 | | MNDA | | 0.720435 | | 0 | |
| 31 | | NCF2 | | 0.715055 | | 0 | |
| 32 | | FYB1 | | 0.714958 | | 0 | |
| 33 | | STAT1 | | 0.712137 | | 0 | |
| 34 | | IL10 | | 0.711394 | | 0 | |
| 35 | | ICOS | | 0.710425 | | 0 | |
| 36 | | SIGLEC7 | | 0.710086 | | 0 | |
| 37 | | CCR8 | | 0.709617 | | 0 | |
| 38 | | PTPN22 | | 0.708095 | | 0 | |
| 39 | | LAIR1 | | 0.705026 | | 0 | |
| 40 | | IL2RA | | 0.704828 | | 0 | |
| 41 | | GBP1 | | 0.70333 | | 0 | |
| 42 | | GPR65 | | 0.70306 | | 0 | |
| 43 | | AIM2 | | 0.701967 | | 0 | |
| 44 | | TNFRSF9 | | 0.701176 | | 0 | |
| 45 | | CXorf21 | | 0.700877 | | 0 | |
| 46 | | CXCL11 | | 0.697867 | | 0 | |
| 47 | | DOCK2 | | 0.69526 | | 0 | |
| 48 | | SAMHD1 | | 0.693488 | | 0 | |
| 49 | | CCL8 | | 0.69244 | | 0 | |
| 50 | | PTPRC | | 0.690867 | | 0 | |
| 51 | | LAPTM5 | | 0.690467 | | 0 | |
| 52 | | SLAMF8 | | 0.69039 | | 0 | |
| 53 | | C3AR1 | | 0.688829 | | 0 | |
| 54 | | 1-Mar | | 0.688205 | | 0 | |
| 55 | | NCKAP1L | | 0.687373 | | 0 | |
| 56 | | FCER1G | | 0.686521 | | 0 | |
| 57 | | LILRA6 | | 0.681954 | | 0 | |
| 58 | | SNX20 | | 0.68175 | | 0 | |
| 59 | | CCR5 | | 0.680788 | | 0 | |
| 60 | | MSR1 | | 0.67982 | | 0 | |
| 61 | | FCGR1A | | 0.679503 | | 0 | |
| 62 | | TMEM150B | | 0.678954 | | 0 | |
| 63 | | CD4 | | 0.678562 | | 0 | |
| 64 | | CTLA4 | | 0.675764 | | 0 | |
| 65 | | ADAMDEC1 | | 0.674545 | | 0 | |
| 66 | | IFIT3 | | 0.673978 | | 0 | |
| 67 | | FPR2 | | 0.673075 | | 0 | |
| 68 | | SIGLEC9 | | 0.672246 | | 0 | |
| 69 | | GPR84 | | 0.671793 | | 0 | |
| 70 | | BTK | | 0.670569 | | 0 | |
| 71 | | TNFSF8 | | 0.670261 | | 0 | |
| 72 | | PILRA | | 0.668519 | | 0 | |
| 73 | | CTSS | | 0.668488 | | 0 | |
| 74 | | KLHL6 | | 0.667499 | | 0 | |
| 75 | | IL21R | | 0.666021 | | 0 | |
| 76 | | CD180 | | 0.664407 | | 0 | |
| 77 | | SIGLEC10 | | 0.664351 | | 0 | |
| 78 | | SCIMP | | 0.662655 | | 0 | |
| 79 | | LILRA5 | | 0.662462 | | 0 | |
| 80 | | IFI44L | | 0.661836 | | 0 | |
| 81 | | MX2 | | 0.661469 | | 0 | |
| 82 | | CMPK2 | | 0.661142 | | 0 | |
| 83 | | ITGAX | | 0.658962 | | 0 | |
| 84 | | SLA | | 0.65809 | | 0 | |
| 85 | | NLRC5 | | 0.657514 | | 0 | |
| 86 | | CD274 | | 0.654401 | | 0 | |
| 87 | | IL4I1 | | 0.65333 | | 0 | |
| 88 | | NLRC4 | | 0.652892 | | 0 | |
| 89 | | PIK3R5 | | 0.651147 | | 0 | |
| 90 | | CD163 | | 0.649763 | | 0 | |
| 91 | | BCL2A1 | | 0.649133 | | 0 | |
| 92 | | IL12RB1 | | 0.648512 | | 0 | |
| 93 | | PARP14 | | 0.648214 | | 0 | |
| 94 | | SLC15A3 | | 0.648175 | | 0 | |
| 95 | | CD300LF | | 0.646538 | | 0 | |
| 96 | | ZBP1 | | 0.646027 | | 0 | |
| 97 | | BIN2 | | 0.645915 | | 0 | |
| 98 | | B2M | | 0.645578 | | 0 | |
| 99 | | TAGAP | | 0.644596 | | 0 | |
| 100 | | TLR8 | | 0.644319 | | 0 | |
| 101 | | OLR1 | | 0.644291 | | 0 | |
| 102 | | TRIM22 | | 0.643954 | | 0 | |
| 103 | | LYN | | 0.643055 | | 0 | |
| 104 | | CHST11 | | 0.642594 | | 0 | |
| 105 | | APOBEC3A | | 0.642117 | | 0 | |
| 106 | | FLVCR2 | | 0.642108 | | 0 | |
| 107 | | SRGN | | 0.641678 | | 0 | |
| 108 | | CD83 | | 0.641361 | | 0 | |
| 109 | | ADGRE2 | | 0.640714 | | 0 | |
| 110 | | ADAP2 | | 0.640301 | | 0 | |
| 111 | | IFIH1 | | 0.640293 | | 0 | |
| 112 | | OAS2 | | 0.639216 | | 0 | |
| 113 | | HLA-DMB | | 0.63919 | | 0 | |
| 114 | | XAF1 | | 0.639039 | | 0 | |
| 115 | | MILR1 | | 0.638389 | | 0 | |
| 116 | | LILRB3 | | 0.637547 | | 0 | |
| 117 | | C1QC | | 0.63575 | | 0 | |
| 118 | | EVI2B | | 0.635466 | | 0 | |
| 119 | | HCK | | 0.635404 | | 0 | |
| 120 | | IL10RA | | 0.635061 | | 0 | |
| 121 | | IGSF6 | | 0.634034 | | 0 | |
| 122 | | IFI44 | | 0.633954 | | 0 | |
| 123 | | ZBED2 | | 0.631714 | | 0 | |
| 124 | | IFIT2 | | 0.631452 | | 0 | |
| 125 | | LPXN | | 0.631185 | | 0 | |
| 126 | | TIGIT | | 0.63074 | | 0 | |
| 127 | | SASH3 | | 0.629997 | | 0 | |
| 128 | | ITGB2 | | 0.628513 | | 0 | |
| 129 | | FCGR2A | | 0.626885 | | 0 | |
| 130 | | FGD2 | | 0.625896 | | 0 | |
| 131 | | CD226 | | 0.625808 | | 0 | |
| 132 | | TLR1 | | 0.625217 | | 0 | |
| 133 | | SIRPB2 | | 0.625216 | | 0 | |
| 134 | | C5AR1 | | 0.624728 | | 0 | |
| 135 | | GBP4 | | 0.623722 | | 0 | |
| 136 | | PIK3CG | | 0.623192 | | 0 | |
| 137 | | C1QB | | 0.6229 | | 0 | |
| 138 | | HK3 | | 0.622548 | | 0 | |
| 139 | | SAMD9L | | 0.621935 | | 0 | |
| 140 | | ST8SIA4 | | 0.619842 | | 0 | |
| 141 | | FOXP3 | | 0.618776 | | 0 | |
| 142 | | FCGR1B | | 0.618536 | | 0 | |
| 143 | | IKZF1 | | 0.618526 | | 0 | |
| 144 | | APOL6 | | 0.617576 | | 0 | |
| 145 | | CASP5 | | 0.617408 | | 0 | |
| 146 | | CD300E | | 0.61663 | | 0 | |
| 147 | | LRRC25 | | 0.615691 | | 0 | |
| 148 | | MS4A6A | | 0.615167 | | 0 | |
| 149 | | IDO1 | | 0.614908 | | 0 | |
| 150 | | PARP15 | | 0.614835 | | 0 | |
| 151 | | TAP1 | | 0.614575 | | 0 | |
| 152 | | WIPF1 | | 0.614457 | | 0 | |
| 153 | | DOK3 | | 0.612708 | | 0 | |
| 154 | | LYZ | | 0.612161 | | 0 | |
| 155 | | THEMIS2 | | 0.611545 | | 0 | |
| 156 | | NCF1 | | 0.610306 | | 0 | |
| 157 | | MPEG1 | | 0.607684 | | 0 | |
| 158 | | SLFN12L | | 0.607188 | | 0 | |
| 159 | | KBTBD8 | | 0.604811 | | 0 | |
| 160 | | PTPN7 | | 0.604558 | | 0 | |
| 161 | | ZC3H12D | | 0.604447 | | 0 | |
| 162 | | LTA | | 0.604394 | | 0 | |
| 163 | | TLR7 | | 0.604046 | | 0 | |
| 164 | | C2 | | 0.603373 | | 0 | |
| 165 | | OSCAR | | 0.602854 | | 0 | |
| 166 | | PTAFR | | 0.602167 | | 0 | |
| 167 | | RASSF4 | | 0.601974 | | 0 | |
| 168 | | MS4A4A | | 0.601531 | | 0 | |
| 169 | | SLAMF7 | | 0.601165 | | 0 | |
| 170 | | CD300A | | 0.600909 | | 0 | |
| 171 | | ARHGAP30 | | 0.600261 | | 0 | |
| 172 | | IRF8 | | 0.599819 | | 0 | |
| 173 | | CD300C | | 0.59981 | | 0 | |
| 174 | | HTRA4 | | 0.599159 | | 0 | |
| 175 | | CD72 | | 0.598852 | | 0 | |
| 176 | | HLA-DRA | | 0.598422 | | 0 | |
| 177 | | HLA-DQA1 | | 0.597875 | | 0 | |
| 178 | | NPL | | 0.597171 | | 0 | |
| 179 | | VCAM1 | | 0.596751 | | 0 | |
| 180 | | OASL | | 0.596593 | | 0 | |
| 181 | | CXCR6 | | 0.59485 | | 0 | |
| 182 | | PLSCR1 | | 0.594801 | | 0 | |
| 183 | | FERMT3 | | 0.594719 | | 0 | |
| 184 | | LAG3 | | 0.594683 | | 0 | |
| 185 | | CYTH4 | | 0.592961 | | 0 | |
| 186 | | APBB1IP | | 0.59292 | | 0 | |
| 187 | | SIGLEC14 | | 0.592742 | | 0 | |
| 188 | | ITGB7 | | 0.592708 | | 0 | |
| 189 | | RAB39A | | 0.592686 | | 0 | |
| 190 | | SIRPB1 | | 0.592639 | | 0 | |
| 191 | | SLC8A1 | | 0.592633 | | 0 | |
| 192 | | SLC1A3 | | 0.592323 | | 0 | |
| 193 | | TMEM52B | | 0.592269 | | 0 | |
| 194 | | SERPINB9 | | 0.592253 | | 0 | |
| 195 | | SUCNR1 | | 0.591593 | | 0 | |
| 196 | | P2RX7 | | 0.590801 | | 0 | |
| 197 | | TRAF3IP3 | | 0.589025 | | 0 | |
| 198 | | DLEU7 | | 0.588897 | | 0 | |
| 199 | | WDFY4 | | 0.587268 | | 0 | |
| 200 | | ADCY7 | | 0.584854 | | 0 | |
| 201 | | TNIP3 | | 0.584725 | | 0 | |
| 202 | | CD38 | | 0.58387 | | 0 | |
| 203 | | LPAR5 | | 0.583688 | | 0 | |
| 204 | | SPN | | 0.582419 | | 0 | |
| 205 | | FGL2 | | 0.582353 | | 0 | |
| 206 | | LGMN | | 0.582284 | | 0 | |
| 207 | | CD28 | | 0.582154 | | 0 | |
| 208 | | TRPV2 | | 0.581041 | | 0 | |
| 209 | | AOAH | | 0.580291 | | 0 | |
| 210 | | CCRL2 | | 0.580241 | | 0 | |
| 211 | | SPI1 | | 0.580201 | | 0 | |
| 212 | | ITGA4 | | 0.579974 | | 0 | |
| 213 | | ARHGAP25 | | 0.579503 | | 0 | |
| 214 | | IL2RG | | 0.578815 | | 0 | |
| 215 | | XIRP1 | | 0.578644 | | 0 | |
| 216 | | CLEC4A | | 0.576504 | | 0 | |
| 217 | | SLCO2B1 | | 0.576374 | | 0 | |
| 218 | | STX11 | | 0.576146 | | 0 | |
| 219 | | EMILIN2 | | 0.575236 | | 0 | |
| 220 | | OAS3 | | 0.574173 | | 0 | |
| 221 | | CTSL | | 0.573973 | | 0 | |
| 222 | | STAT2 | | 0.573318 | | 0 | |
| 223 | | EVI2A | | 0.573268 | | 0 | |
| 224 | | UBASH3B | | 0.572449 | | 0 | |
| 225 | | TIMD4 | | 0.571741 | | 0 | |
| 226 | | ADAM19 | | 0.570166 | | 0 | |
| 227 | | PLA2G7 | | 0.569915 | | 0 | |
| 228 | | SLC2A5 | | 0.569528 | | 0 | |
| 229 | | CALHM6 | | 0.569368 | | 0 | |
| 230 | | PAG1 | | 0.569254 | | 0 | |
| 231 | | CRTAM | | 0.567999 | | 0 | |
| 232 | | CLEC6A | | 0.567897 | | 0 | |
| 233 | | FPR1 | | 0.567588 | | 0 | |
| 234 | | TAP2 | | 0.566907 | | 0 | |
| 235 | | IL2RB | | 0.566757 | | 0 | |
| 236 | | CCL4 | | 0.564908 | | 0 | |
| 237 | | SLA2 | | 0.564786 | | 0 | |
| 238 | | CXCL9 | | 0.563522 | | 0 | |
| 239 | | TLR4 | | 0.56296 | | 0 | |
| 240 | | TLR2 | | 0.561737 | | 0 | |
| 241 | | CD48 | | 0.561322 | | 0 | |
| 242 | | ETV7 | | 0.561078 | | 0 | |
| 243 | | CCR4 | | 0.561078 | | 0 | |
| 244 | | FMNL1 | | 0.56035 | | 0 | |
| 245 | | CR1L | | 0.56034 | | 0 | |
| 246 | | P2RY6 | | 0.560147 | | 0 | |
| 247 | | MYO1G | | 0.559868 | | 0 | |
| 248 | | MX1 | | 0.558937 | | 0 | |
| 249 | | FAM78A | | 0.558878 | | 0 | |
| 250 | | GZMB | | 0.558675 | | 0 | |
| 251 | | LAMP3 | | 0.558541 | | 0 | |
| 252 | | ZNF804A | | 0.55708 | | 0 | |
| 253 | | FASLG | | 0.557017 | | 0 | |
| 254 | | LAP3 | | 0.556038 | | 0 | |
| 255 | | CD2 | | 0.555982 | | 0 | |
| 256 | | IFNG | | 0.555902 | | 0 | |
| 257 | | CD96 | | 0.555731 | | 0 | |
| 258 | | AQP9 | | 0.555009 | | 0 | |
| 259 | | CARD17 | | 0.554811 | | 0 | |
| 260 | | TRIM69 | | 0.554627 | | 0 | |
| 261 | | ITK | | 0.554435 | | 0 | |
| 262 | | CARD11 | | 0.553948 | | 0 | |
| 263 | | IL18BP | | 0.553718 | | 0 | |
| 264 | | IFI30 | | 0.553588 | | 0 | |
| 265 | | SELPLG | | 0.552725 | | 0 | |
| 266 | | MYO1F | | 0.551976 | | 0 | |
| 267 | | HERC5 | | 0.550088 | | 0 | |
| 268 | | PSMB9 | | 0.549919 | | 0 | |
| 269 | | SIRPG | | 0.549292 | | 0 | |
| 270 | | CD33 | | 0.549247 | | 0 | |
| 271 | | APOL1 | | 0.548767 | | 0 | |
| 272 | | KLRD1 | | 0.548101 | | 0 | |
| 273 | | GPR31 | | 0.547593 | | 0 | |
| 274 | | WARS | | 0.546839 | | 0 | |
| 275 | | VNN2 | | 0.546736 | | 0 | |
| 276 | | CD3G | | 0.546596 | | 0 | |
| 277 | | GPR174 | | 0.54655 | | 0 | |
| 278 | | F5 | | 0.546483 | | 0 | |
| 279 | | MPP1 | | 0.546234 | | 0 | |
| 280 | | DSE | | 0.546044 | | 0 | |
| 281 | | SAMD9 | | 0.545806 | | 0 | |
| 282 | | ACOD1 | | 0.545413 | | 0 | |
| 283 | | ITGAM | | 0.545222 | | 0 | |
| 284 | | RNASE6 | | 0.544711 | | 0 | |
| 285 | | CSF1R | | 0.544646 | | 0 | |
| 286 | | GNGT2 | | 0.543878 | | 0 | |
| 287 | | BATF2 | | 0.543145 | | 0 | |
| 288 | | PATL2 | | 0.543096 | | 0 | |
| 289 | | TNFSF14 | | 0.543065 | | 0 | |
| 290 | | C1orf162 | | 0.542946 | | 0 | |
| 291 | | PYHIN1 | | 0.542845 | | 0 | |
| 292 | | LHFPL2 | | 0.542762 | | 0 | |
| 293 | | ARHGAP9 | | 0.542021 | | 0 | |
| 294 | | MCOLN2 | | 0.541943 | | 0 | |
| 295 | | LAX1 | | 0.541522 | | 0 | |
| 296 | | CTSC | | 0.54139 | | 0 | |
| 297 | | CD37 | | 0.541042 | | 0 | |
| 298 | | IFI16 | | 0.539997 | | 0 | |
| 299 | | STAP1 | | 0.539881 | | 0 | |
| 300 | | CCL7 | | 0.539744 | | 0 | |
| 301 | | TMEM140 | | 0.538507 | | 0 | |
| 302 | | NCF4 | | 0.538151 | | 0 | |
| 303 | | CIITA | | 0.537839 | | 0 | |
| 304 | | TNFAIP8L2 | | 0.537724 | | 0 | |
| 305 | | SAMD3 | | 0.537721 | | 0 | |
| 306 | | CD74 | | 0.537393 | | 0 | |
| 307 | | KIR2DL4 | | 0.53739 | | 0 | |
| 308 | | PLEKHO2 | | 0.537329 | | 0 | |
| 309 | | C1QA | | 0.537077 | | 0 | |
| 310 | | P2RY10 | | 0.535647 | | 0 | |
| 311 | | ALOX5AP | | 0.535283 | | 0 | |
| 312 | | TNFAIP3 | | 0.534728 | | 0 | |
| 313 | | LAT2 | | 0.533831 | | 0 | |
| 314 | | BCAT1 | | 0.533411 | | 0 | |
| 315 | | FGR | | 0.533273 | | 0 | |
| 316 | | PRDM1 | | 0.532749 | | 0 | |
| 317 | | DAPK1 | | 0.531769 | | 0 | |
| 318 | | CYTIP | | 0.531013 | | 0 | |
| 319 | | SLAMF6 | | 0.529943 | | 0 | |
| 320 | | CASP1 | | 0.529566 | | 0 | |
| 321 | | BTN3A1 | | 0.529379 | | 0 | |
| 322 | | GAB3 | | 0.528893 | | 0 | |
| 323 | | RAB42 | | 0.528681 | | 0 | |
| 324 | | SH2D1A | | 0.528067 | | 0 | |
| 325 | | HLA-DOA | | 0.528006 | | 0 | |
| 326 | | HCLS1 | | 0.52786 | | 0 | |
| 327 | | SLAMF1 | | 0.527709 | | 0 | |
| 328 | | STAT4 | | 0.527693 | | 0 | |
| 329 | | ADA2 | | 0.527574 | | 0 | |
| 330 | | HLA-DPA1 | | 0.526487 | | 0 | |
| 331 | | GPR18 | | 0.523998 | | 0 | |
| 332 | | MEFV | | 0.523793 | | 0 | |
| 333 | | IL15RA | | 0.523504 | | 0 | |
| 334 | | FMNL2 | | 0.522747 | | 0 | |
| 335 | | HLA-DRB1 | | 0.522712 | | 0 | |
| 336 | | VAV1 | | 0.522304 | | 0 | |
| 337 | | MLKL | | 0.522218 | | 0 | |
| 338 | | RAP2B | | 0.522106 | | 0 | |
| 339 | | CCL2 | | 0.521903 | | 0 | |
| 340 | | PARP9 | | 0.521349 | | 0 | |
| 341 | | TIFAB | | 0.521264 | | 0 | |
| 342 | | CASS4 | | 0.521004 | | 0 | |
| 343 | | GPR183 | | 0.520904 | | 0 | |
| 344 | | WAS | | 0.520503 | | 0 | |
| 345 | | KCNA3 | | 0.520464 | | 0 | |
| 346 | | TLDC2 | | 0.519883 | | 0 | |
| 347 | | FCAR | | 0.518857 | | 0 | |
| 348 | | BTN3A3 | | 0.518747 | | 0 | |
| 349 | | CD5 | | 0.518148 | | 0 | |
| 350 | | HLA-B | | 0.518077 | | 0 | |
| 351 | | LAIR2 | | 0.517964 | | 0 | |
| 352 | | BIRC3 | | 0.517875 | | 0 | |
| 353 | | CST7 | | 0.517874 | | 0 | |
| 354 | | PLCB2 | | 0.517494 | | 0 | |
| 355 | | CPVL | | 0.517323 | | 0 | |
| 356 | | IRF1 | | 0.517229 | | 0 | |
| 357 | | GPR141 | | 0.51696 | | 0 | |
| 358 | | TYROBP | | 0.516731 | | 0 | |
| 359 | | ICAM1 | | 0.516561 | | 0 | |
| 360 | | CSF2RB | | 0.516498 | | 0 | |
| 361 | | APOBEC3H | | 0.516337 | | 0 | |
| 362 | | IL15 | | 0.5163 | | 0 | |
| 363 | | AIF1 | | 0.516284 | | 0 | |
| 364 | | NFAM1 | | 0.516261 | | 0 | |
| 365 | | SIGLEC5 | | 0.516057 | | 0 | |
| 366 | | HLA-DMA | | 0.515832 | | 0 | |
| 367 | | PARVG | | 0.51561 | | 0 | |
| 368 | | MERTK | | 0.515599 | | 0 | |
| 369 | | IGF2BP3 | | 0.514805 | | 0 | |
| 370 | | TLR6 | | 0.5134 | | 0 | |
| 371 | | CCL5 | | 0.513216 | | 0 | |
| 372 | | CSF2RA | | 0.512596 | | 0 | |
| 373 | | LGALS9 | | 0.511944 | | 0 | |
| 374 | | ARHGAP15 | | 0.511057 | | 0 | |
| 375 | | GTSF1 | | 0.510736 | | 0 | |
| 376 | | CD200R1 | | 0.509826 | | 0 | |
| 377 | | GPNMB | | 0.509593 | | 0 | |
| 378 | | PRKCB | | 0.509417 | | 0 | |
| 379 | | SDS | | 0.50918 | | 0 | |
| 380 | | NMI | | 0.508289 | | 0 | |
| 381 | | IRF4 | | 0.507766 | | 0 | |
| 382 | | STARD4 | | 0.507132 | | 0 | |
| 383 | | GRAMD1B | | 0.506915 | | 0 | |
| 384 | | KCNAB2 | | 0.50525 | | 0 | |
| 385 | | RASGEF1B | | 0.505192 | | 0 | |
| 386 | | RNF19B | | 0.505074 | | 0 | |
| 387 | | OSM | | 0.504752 | | 0 | |
| 388 | | EPHB2 | | 0.504563 | | 0 | |
| 389 | | TRAT1 | | 0.504458 | | 0 | |
| 390 | | SSTR3 | | 0.504392 | | 0 | |
| 391 | | IL1B | | 0.503898 | | 0 | |
| 392 | | CXCR3 | | 0.503486 | | 0 | |
| 393 | | ADGRE1 | | 0.503387 | | 0 | |
| 394 | | SP100 | | 0.503278 | | 0 | |
| 395 | | DDX60 | | 0.501876 | | 0 | |
| 396 | | IL18RAP | | 0.500355 | | 0 | |
| 397 | | IFI27 | | 0.499773 | | 0 | |
| 398 | | CORO1C | | 0.499202 | | 0 | |
| 399 | | DOCK11 | | 0.499194 | | 0 | |
| 400 | | CCR2 | | 0.499137 | | 0 | |
| 401 | | PRF1 | | 0.498631 | | 0 | |
| 402 | | SIRPD | | 0.498347 | | 0 | |
| 403 | | LCP1 | | 0.498029 | | 0 | |
| 404 | | DDX58 | | 0.497883 | | 0 | |
| 405 | | IL7R | | 0.496588 | | 0 | |
| 406 | | BTLA | | 0.496103 | | 0 | |
| 407 | | DOK2 | | 0.495995 | | 0 | |
| 408 | | UBASH3A | | 0.49514 | | 0 | |
| 409 | | KLHDC7B | | 0.495078 | | 0 | |
| 410 | | RUBCNL | | 0.494714 | | 0 | |
| 411 | | CD6 | | 0.49433 | | 0 | |
| 412 | | GPR171 | | 0.493889 | | 0 | |
| 413 | | TBX21 | | 0.49376 | | 0 | |
| 414 | | CLEC4E | | 0.493392 | | 0 | |
| 415 | | USP18 | | 0.492746 | | 0 | |
| 416 | | ARSB | | 0.492533 | | 0 | |
| 417 | | STK17B | | 0.492239 | | 0 | |
| 418 | | DOCK8 | | 0.492233 | | 0 | |
| 419 | | TNFRSF1B | | 0.491839 | | 0 | |
| 420 | | ACSL4 | | 0.491577 | | 0 | |
| 421 | | LY96 | | 0.491432 | | 0 | |
| 422 | | EIF2AK2 | | 0.491033 | | 0 | |
| 423 | | IFIT1 | | 0.490679 | | 0 | |
| 424 | | ACTR3 | | 0.490183 | | 0 | |
| 425 | | THEMIS | | 0.490078 | | 0 | |
| 426 | | SOD2 | | 0.489298 | | 0 | |
| 427 | | STK17A | | 0.489126 | | 0 | |
| 428 | | CCL4L2 | | 0.488841 | | 0 | |
| 429 | | RUNX3 | | 0.488447 | | 0 | |
| 430 | | KYNU | | 0.48756 | | 0 | |
| 431 | | PLAUR | | 0.487559 | | 0 | |
| 432 | | KCNJ10 | | 0.487321 | | 0 | |
| 433 | | LCK | | 0.486588 | | 0 | |
| 434 | | SLFN11 | | 0.486511 | | 0 | |
| 435 | | APOBEC3G | | 0.485647 | | 0 | |
| 436 | | FCGR2B | | 0.485457 | | 0 | |
| 437 | | ZNF831 | | 0.485288 | | 0 | |
| 438 | | CTSB | | 0.48471 | | 0 | |
| 439 | | CD3D | | 0.48429 | | 0 | |
| 440 | | CD3E | | 0.484137 | | 0 | |
| 441 | | PRKCQ | | 0.48393 | | 0 | |
| 442 | | RUFY4 | | 0.483902 | | 0 | |
| 443 | | ADGRE5 | | 0.483622 | | 0 | |
| 444 | | TEX11 | | 0.483581 | | 0 | |
| 445 | | ANKRD44 | | 0.482953 | | 0 | |
| 446 | | SH2D2A | | 0.482815 | | 0 | |
| 447 | | GNLY | | 0.482781 | | 0 | |
| 448 | | C15orf53 | | 0.482767 | | 0 | |
| 449 | | PIK3CD | | 0.482512 | | 0 | |
| 450 | | CLECL1 | | 0.482269 | | 0 | |
| 451 | | LY86 | | 0.481973 | | 0 | |
| 452 | | COL6A5 | | 0.481741 | | 0 | |
| 453 | | STK4 | | 0.481498 | | 0 | |
| 454 | | HERC6 | | 0.481304 | | 0 | |
| 455 | | INPP5D | | 0.481235 | | 0 | |
| 456 | | PLXNC1 | | 0.481073 | | 0 | |
| 457 | | ZNF683 | | 0.480827 | | 0 | |
| 458 | | HMOX1 | | 0.47967 | | 0 | |
| 459 | | PARP12 | | 0.479521 | | 0 | |
| 460 | | GFI1 | | 0.479413 | | 0 | |
| 461 | | NABP1 | | 0.479341 | | 0 | |
| 462 | | C16orf54 | | 0.478865 | | 0 | |
| 463 | | TM6SF1 | | 0.478757 | | 0 | |
| 464 | | PDCD1 | | 0.478722 | | 0 | |
| 465 | | FCRL3 | | 0.478564 | | 0 | |
| 466 | | CD247 | | 0.478089 | | 0 | |
| 467 | | CCL13 | | 0.4778 | | 0 | |
| 468 | | PSTPIP1 | | 0.477642 | | 0 | |
| 469 | | CMKLR1 | | 0.476801 | | 0 | |
| 470 | | DOCK10 | | 0.476447 | | 0 | |
| 471 | | IKZF3 | | 0.476263 | | 0 | |
| 472 | | ZNFX1 | | 0.475965 | | 0 | |
| 473 | | GBP6 | | 0.47561 | | 0 | |
| 474 | | ADAM8 | | 0.475498 | | 0 | |
| 475 | | DNAJC5B | | 0.47544 | | 0 | |
| 476 | | EOMES | | 0.475412 | | 0 | |
| 477 | | NT5E | | 0.474513 | | 0 | |
| 478 | | TMC8 | | 0.473476 | | 0 | |
| 479 | | SIRPA | | 0.473407 | | 0 | |
| 480 | | SELL | | 0.47325 | | 0 | |
| 481 | | JAK3 | | 0.473228 | | 0 | |
| 482 | | GZMA | | 0.473187 | | 0 | |
| 483 | | SLC24A4 | | 0.472176 | | 0 | |
| 484 | | TREML2 | | 0.472123 | | 0 | |
| 485 | | ICAM3 | | 0.471811 | | 0 | |
| 486 | | PPP1R18 | | 0.471411 | | 0 | |
| 487 | | CLEC12A | | 0.470729 | | 0 | |
| 488 | | SLC6A12 | | 0.469576 | | 0 | |
| 489 | | CLNK | | 0.469341 | | 0 | |
| 490 | | C19orf84 | | 0.468779 | | 0 | |
| 491 | | GAL3ST4 | | 0.468335 | | 0 | |
| 492 | | HS3ST3B1 | | 0.468329 | | 0 | |
| 493 | | SIT1 | | 0.46739 | | 0 | |
| 494 | | GIMAP4 | | 0.466788 | | 0 | |
| 495 | | SP110 | | 0.466523 | | 0 | |
| 496 | | IL1A | | 0.466268 | | 0 | |
| 497 | | TNFSF4 | | 0.466108 | | 0 | |
| 498 | | CORO1A | | 0.464422 | | 0 | |
| 499 | | CARD16 | | 0.464367 | | 0 | |
| 500 | | TNFAIP8 | | 0.464045 | | 0 | |
| 501 | | IL12RB2 | | 0.463928 | | 0 | |
| 502 | | LST1 | | 0.463725 | | 0 | |
| 503 | | TGFBI | | 0.463458 | | 0 | |
| 504 | | GLT1D1 | | 0.463308 | | 0 | |
| 505 | | POU2F2 | | 0.462793 | | 0 | |
| 506 | | UBD | | 0.462647 | | 0 | |
| 507 | | LY9 | | 0.461748 | | 0 | |
| 508 | | CD7 | | 0.461523 | | 0 | |
| 509 | | ZNF80 | | 0.461105 | | 0 | |
| 510 | | UBE2L6 | | 0.460968 | | 0 | |
| 511 | | ITGAL | | 0.460886 | | 0 | |
| 512 | | FOXN2 | | 0.460855 | | 0 | |
| 513 | | JAKMIP2 | | 0.460842 | | 0 | |
| 514 | | MAP4K1 | | 0.460692 | | 0 | |
| 515 | | SGTB | | 0.46044 | | 0 | |
| 516 | | CELF2 | | 0.459821 | | 0 | |
| 517 | | CD40 | | 0.459772 | | 0 | |
| 518 | | MSN | | 0.459339 | | 0 | |
| 519 | | SPOCK2 | | 0.459138 | | 0 | |
| 520 | | HLA-DRB5 | | 0.458817 | | 0 | |
| 521 | | IL9R | | 0.458761 | | 0 | |
| 522 | | DTX3L | | 0.458756 | | 0 | |
| 523 | | NCR1 | | 0.457695 | | 0 | |
| 524 | | CLEC4D | | 0.457577 | | 0 | |
| 525 | | HLA-F | | 0.45703 | | 0 | |
| 526 | | RASGRP3 | | 0.456191 | | 0 | |
| 527 | | CD244 | | 0.455944 | | 0 | |
| 528 | | BTN2A2 | | 0.455565 | | 0 | |
| 529 | | DAPP1 | | 0.455241 | | 0 | |
| 530 | | RGS18 | | 0.454414 | | 0 | |
| 531 | | GLIPR1 | | 0.45433 | | 0 | |
| 532 | | LACC1 | | 0.454037 | | 0 | |
| 533 | | GTSF1L | | 0.453755 | | 0 | |
| 534 | | SLCO5A1 | | 0.453246 | | 0 | |
| 535 | | CLEC4C | | 0.453194 | | 0 | |
| 536 | | TRIM21 | | 0.452893 | | 0 | |
| 537 | | NKG7 | | 0.452644 | | 0 | |
| 538 | | CD8A | | 0.452624 | | 0 | |
| 539 | | DDX60L | | 0.452553 | | 0 | |
| 540 | | ZEB2 | | 0.451454 | | 0 | |
| 541 | | PTGER4 | | 0.451016 | | 0 | |
| 542 | | CCL3 | | 0.450356 | | 0 | |
| 543 | | TTC24 | | 0.450045 | | 0 | |
| 544 | | BTN3A2 | | 0.450011 | | 0 | |
| 545 | | HLA-DQB1 | | 0.449502 | | 0 | |
| 546 | | OPTN | | 0.449017 | | 0 | |
| 547 | | EML4 | | 0.448962 | | 0 | |
| 548 | | TESPA1 | | 0.448926 | | 0 | |
| 549 | | XCL1 | | 0.448783 | | 0 | |
| 550 | | CD52 | | 0.448512 | | 0 | |
| 551 | | IL21 | | 0.447045 | | 0 | |
| 552 | | EGFL6 | | 0.446936 | | 0 | |
| 553 | | HLA-A | | 0.446559 | | 0 | |
| 554 | | CR1 | | 0.446495 | | 0 | |
| 555 | | HLA-E | | 0.44625 | | 0 | |
| 556 | | RASSF2 | | 0.446071 | | 0 | |
| 557 | | SLC9A9 | | 0.446056 | | 0 | |
| 558 | | IL16 | | 0.445646 | | 0 | |
| 559 | | TREM1 | | 0.445583 | | 0 | |
| 560 | | KLRC1 | | 0.445206 | | 0 | |
| 561 | | ALPK2 | | 0.445091 | | 0 | |
| 562 | | ARNTL2 | | 0.444567 | | 0 | |
| 563 | | CERKL | | 0.444507 | | 0 | |
| 564 | | CD69 | | 0.444357 | | 0 | |
| 565 | | SCML4 | | 0.444247 | | 0 | |
| 566 | | NUGGC | | 0.444228 | | 0 | |
| 567 | | IFNAR2 | | 0.444183 | | 0 | |
| 568 | | OTOF | | 0.443457 | | 0 | |
| 569 | | NLRP3 | | 0.443404 | | 0 | |
| 570 | | FAM20A | | 0.443401 | | 0 | |
| 571 | | MTHFD1L | | 0.443383 | | 0 | |
| 572 | | GTF2IRD2 | | -0.44306 | | 0 | |
| 573 | | IFIT5 | | 0.443007 | | 0 | |
| 574 | | LIMS1 | | 0.442963 | | 0 | |
| 575 | | RCSD1 | | 0.440975 | | 0 | |
| 576 | | HVCN1 | | 0.440913 | | 0 | |
| 577 | | CYLD | | 0.440777 | | 0 | |
| 578 | | MYD88 | | 0.44062 | | 0 | |
| 579 | | APOC1 | | 0.440211 | | 0 | |
| 580 | | IL32 | | 0.438825 | | 0 | |
| 581 | | PLA2G2D | | 0.438809 | | 0 | |
| 582 | | SLC11A1 | | 0.438781 | | 0 | |
| 583 | | C1S | | 0.438749 | | 0 | |
| 584 | | C5orf58 | | 0.43835 | | 0 | |
| 585 | | ARPC2 | | 0.438341 | | 0 | |
| 586 | | GPR82 | | 0.437978 | | 0 | |
| 587 | | CCL11 | | 0.437867 | | 0 | |
| 588 | | CCNYL1 | | 0.437777 | | 0 | |
| 589 | | ARL4C | | 0.437398 | | 0 | |
| 590 | | IL12B | | 0.437385 | | 0 | |
| 591 | | XCL2 | | 0.437228 | | 0 | |
| 592 | | GTF2IRD2B | | -0.43691 | | 0 | |
| 593 | | VNN1 | | 0.43669 | | 0 | |
| 594 | | DPEP2 | | 0.436583 | | 0 | |
| 595 | | IL18R1 | | 0.436349 | | 0 | |
| 596 | | MEP1A | | 0.436261 | | 0 | |
| 597 | | CYSLTR2 | | 0.436066 | | 0 | |
| 598 | | VSIG4 | | 0.436013 | | 0 | |
| 599 | | RELT | | 0.435139 | | 0 | |
| 600 | | GCH1 | | 0.434989 | | 0 | |
| 601 | | HLA-DPB1 | | 0.434952 | | 0 | |
| 602 | | RTP4 | | 0.43493 | | 0 | |
| 603 | | ATP6V1B2 | | 0.434111 | | 0 | |
| 604 | | FCGR3B | | 0.434052 | | 0 | |
| 605 | | NCK1 | | 0.433847 | | 0 | |
| 606 | | CD27 | | 0.43351 | | 0 | |
| 607 | | IL7 | | 0.433406 | | 0 | |
| 608 | | OR2I1P | | 0.432993 | | 0 | |
| 609 | | FUT4 | | 0.432168 | | 0 | |
| 610 | | MEI1 | | 0.432131 | | 0 | |
| 611 | | SEMA4D | | 0.43137 | | 0 | |
| 612 | | KIR3DL2 | | 0.431257 | | 0 | |
| 613 | | IFNL1 | | 0.431077 | | 0 | |
| 614 | | IPCEF1 | | 0.431019 | | 0 | |
| 615 | | AXL | | 0.430806 | | 0 | |
| 616 | | SOAT1 | | 0.430133 | | 0 | |
| 617 | | IFI6 | | 0.429947 | | 0 | |
| 618 | | CCDC88A | | 0.429715 | | 0 | |
| 619 | | SEL1L3 | | 0.429448 | | 0 | |
| 620 | | ASAP1 | | 0.429234 | | 0 | |
| 621 | | CIRBP | | -0.42902 | | 0 | |
| 622 | | USP15 | | 0.428811 | | 0 | |
| 623 | | ARAP2 | | 0.428591 | | 0 | |
| 624 | | SERPINB8 | | 0.42827 | | 0 | |
| 625 | | SH2B3 | | 0.427814 | | 0 | |
| 626 | | DGKG | | 0.42747 | | 0 | |
| 627 | | MRC1 | | 0.427462 | | 0 | |
| 628 | | GRAP2 | | 0.427255 | | 0 | |
| 629 | | TDRD6 | | 0.42697 | | 0 | |
| 630 | | LILRA4 | | 0.426242 | | 0 | |
| 631 | | CLEC2B | | 0.426159 | | 0 | |
| 632 | | NRP2 | | 0.425972 | | 0 | |
| 633 | | FUT7 | | 0.425958 | | 0 | |
| 634 | | DRAM1 | | 0.425376 | | 0 | |
| 635 | | ZNF446 | | -0.42497 | | 0 | |
| 636 | | SEMA7A | | 0.424668 | | 0 | |
| 637 | | FAS | | 0.424571 | | 0 | |
| 638 | | WNT2 | | 0.424445 | | 0 | |
| 639 | | PLCL2 | | 0.424394 | | 0 | |
| 640 | | IL18 | | 0.424363 | | 0 | |
| 641 | | CASP10 | | 0.424351 | | 0 | |
| 642 | | TRANK1 | | 0.424246 | | 0 | |
| 643 | | KCNK13 | | 0.42397 | | 0 | |
| 644 | | RRNAD1 | | -0.42287 | | 0 | |
| 645 | | MYO7A | | 0.422862 | | 0 | |
| 646 | | MCEMP1 | | 0.421825 | | 0 | |
| 647 | | TNFSF9 | | 0.421777 | | 0 | |
| 648 | | TIGAR | | 0.421378 | | 0 | |
| 649 | | RAB33A | | 0.421192 | | 0 | |
| 650 | | FFAR4 | | 0.421147 | | 0 | |
| 651 | | PLAU | | 0.420826 | | 0 | |
| 652 | | CGAS | | 0.420731 | | 0 | |
| 653 | | PNOC | | 0.420707 | | 0 | |
| 654 | | ME2 | | 0.420217 | | 0 | |
| 655 | | APOL3 | | 0.419793 | | 0 | |
| 656 | | FAM49B | | 0.419647 | | 0 | |
| 657 | | FAAH | | -0.41954 | | 0 | |
| 658 | | DTHD1 | | 0.419509 | | 0 | |
| 659 | | CXCL13 | | 0.418406 | | 0 | |
| 660 | | CCDC24 | | -0.41835 | | 0 | |
| 661 | | GIMAP5 | | 0.418108 | | 0 | |
| 662 | | STK10 | | 0.417288 | | 0 | |
| 663 | | GAMT | | -0.41691 | | 0 | |
| 664 | | SLC38A5 | | 0.416745 | | 0 | |
| 665 | | ADAM28 | | 0.416162 | | 0 | |
| 666 | | NLRC3 | | 0.416045 | | 0 | |
| 667 | | ZNF875 | | -0.41604 | | 0 | |
| 668 | | OVOL2 | | -0.41573 | | 0 | |
| 669 | | SLFN5 | | 0.415563 | | 0 | |
| 670 | | CD101 | | 0.415163 | | 0 | |
| 671 | | ZNF267 | | 0.4147 | | 0 | |
| 672 | | PTCRA | | 0.414091 | | 0 | |
| 673 | | CCL18 | | 0.413984 | | 0 | |
| 674 | | GM2A | | 0.413145 | | 0 | |
| 675 | | CYB5D2 | | -0.41305 | | 0 | |
| 676 | | TOX | | 0.41296 | | 0 | |
| 677 | | LRMP | | 0.412951 | | 0 | |
| 678 | | ST18 | | 0.412928 | | 0 | |
| 679 | | GZMK | | 0.412716 | | 0 | |
| 680 | | STAMBPL1 | | 0.411785 | | 0 | |
| 681 | | BCAM | | -0.41171 | | 0 | |
| 682 | | TMEM156 | | 0.411183 | | 0 | |
| 683 | | MMP1 | | 0.410949 | | 0 | |
| 684 | | FCRL5 | | 0.410816 | | 0 | |
| 685 | | ZER1 | | -0.41077 | | 0 | |
| 686 | | RAB8B | | 0.410314 | | 0 | |
| 687 | | CD40LG | | 0.410021 | | 0 | |
| 688 | | MIEF2 | | -0.40998 | | 0 | |
| 689 | | AKNA | | 0.409875 | | 0 | |
| 690 | | ZAP70 | | 0.40967 | | 0 | |
| 691 | | CCR7 | | 0.409573 | | 0 | |
| 692 | | PLAC8 | | 0.409433 | | 0 | |
| 693 | | TNFRSF8 | | 0.409406 | | 0 | |
| 694 | | KLRC4-KLRK1 | | 0.409283 | | 0 | |
| 695 | | SYT11 | | 0.40927 | | 0 | |
| 696 | | CAMK4 | | 0.408834 | | 0 | |
| 697 | | OTULIN | | 0.408801 | | 0 | |
| 698 | | NCR3 | | 0.408603 | | 0 | |
| 699 | | TDO2 | | 0.408334 | | 0 | |
| 700 | | GPA33 | | 0.407879 | | 0 | |
| 701 | | DCSTAMP | | 0.407797 | | 0 | |
| 702 | | ABCC4 | | 0.407317 | | 0 | |
| 703 | | RASAL3 | | 0.407194 | | 0 | |
| 704 | | EMP3 | | 0.406994 | | 0 | |
| 705 | | HIVEP2 | | 0.406989 | | 0 | |
| 706 | | ANKRD22 | | 0.406927 | | 0 | |
| 707 | | ADPRH | | 0.406914 | | 0 | |
| 708 | | IRF5 | | 0.406675 | | 0 | |
| 709 | | COTL1 | | 0.406204 | | 0 | |
| 710 | | LILRB5 | | 0.40591 | | 0 | |
| 711 | | HAMP | | 0.405838 | | 0 | |
| 712 | | SIRT3 | | -0.40571 | | 0 | |
| 713 | | B3GNT7 | | 0.405616 | | 0 | |
| 714 | | FBXW4 | | -0.40553 | | 0 | |
| 715 | | VPS37D | | -0.40548 | | 0 | |
| 716 | | TRAF1 | | 0.405472 | | 0 | |
| 717 | | C11orf21 | | 0.405207 | | 0 | |
| 718 | | IDO2 | | 0.404967 | | 0 | |
| 719 | | SLC37A2 | | 0.404027 | | 0 | |
| 720 | | TMIGD3 | | 0.403378 | | 0 | |
| 721 | | SH3KBP1 | | 0.403295 | | 0 | |
| 722 | | PEX11G | | -0.40303 | | 0 | |
| 723 | | ISG15 | | 0.402994 | | 0 | |
| 724 | | ALOX5 | | 0.402647 | | 0 | |
| 725 | | PATL1 | | 0.402515 | | 0 | |
| 726 | | PIM2 | | 0.402478 | | 0 | |
| 727 | | APOL2 | | 0.402382 | | 0 | |
| 728 | | IRF2BP1 | | -0.40185 | | 0 | |
| 729 | | CD8B | | 0.401831 | | 0 | |
| 730 | | CTSW | | 0.401328 | | 0 | |
| 731 | | KCNJ5 | | 0.401038 | | 0 | |
| 732 | | GZMH | | 0.400854 | | 0 | |
| 733 | | AMPD3 | | 0.400697 | | 0 | |
| **Table S2 Genes correlated with CD80 in METABRIC cohort** | | | | | | |  |
|  | symbol | | correlation | | pvalue | |  |
| 1 | IL21R | | 0.427221 | | 2.50E-85 | |  |
| 2 | RASSF4 | | 0.41866 | | 1.12E-81 | |  |
| 3 | DOCK2 | | 0.41785 | | 2.45E-81 | |  |
| 4 | STX11 | | 0.459566 | | 4.11E-100 | |  |
| 5 | WAS | | 0.407612 | | 4.04E-77 | |  |
| 6 | ARHGAP9 | | 0.405263 | | 3.58E-76 | |  |
| 7 | NPL | | 0.47406 | | 2.84E-107 | |  |
| 8 | IL2RA | | 0.436725 | | 1.65E-89 | |  |
| 9 | OSCAR | | 0.439025 | | 1.54E-90 | |  |
| 10 | APOBEC3H | | 0.449707 | | 1.95E-95 | |  |
| 11 | GCH1 | | 0.437572 | | 6.91E-90 | |  |
| 12 | OAS2 | | 0.505518 | | 4.73E-124 | |  |
| 13 | IDO1 | | 0.498673 | | 3.45E-120 | |  |
| 14 | XAF1 | | 0.455507 | | 3.61E-98 | |  |
| 15 | CD53 | | 0.521284 | | 3.81E-133 | |  |
| 16 | FCGR1C | | 0.526403 | | 3.34E-136 | |  |
| 17 | NUP62 | | 0.554027 | | 1.22E-153 | |  |
| 18 | HPSE | | 0.46583 | | 3.65E-103 | |  |
| 19 | IFNG | | 0.404669 | | 6.20E-76 | |  |
| 20 | C2 | | 0.479168 | | 7.03E-110 | |  |
| 21 | APOE | | 0.402685 | | 3.84E-75 | |  |
| 22 | RGS19 | | 0.472924 | | 1.07E-106 | |  |
| 23 | PSME2 | | 0.447239 | | 2.74E-94 | |  |
| 24 | CYBB | | 0.456222 | | 1.65E-98 | |  |
| 25 | HUAT | | 0.489425 | | 3.00E-115 | |  |
| 26 | LGALS9C | | 0.442743 | | 3.18E-92 | |  |
| 27 | IFI6 | | 0.414974 | | 3.88E-80 | |  |
| 28 | LTA | | 0.405253 | | 3.61E-76 | |  |
| 29 | HLA-E | | 0.428656 | | 5.96E-86 | |  |
| 30 | HAMP | | 0.420703 | | 1.54E-82 | |  |
| 31 | APOBEC3G | | 0.419805 | | 3.69E-82 | |  |
| 32 | AIM2 | | 0.505264 | | 6.56E-124 | |  |
| 33 | SAMSN1 | | 0.451632 | | 2.45E-96 | |  |
| 34 | FGL2 | | 0.425139 | | 1.97E-84 | |  |
| 35 | C1QC | | 0.532259 | | 9.16E-140 | |  |
| 36 | RUNX3 | | 0.402757 | | 3.60E-75 | |  |
| 37 | RTP4 | | 0.42891 | | 4.62E-86 | |  |
| 38 | IFIT3 | | 0.485537 | | 3.42E-113 | |  |
| 39 | FLVCR2 | | 0.504183 | | 2.65E-123 | |  |
| 40 | GBP5 | | 0.571093 | | 2.95E-165 | |  |
| 41 | HCST | | 0.402606 | | 4.13E-75 | |  |
| 42 | MX1 | | 0.483048 | | 6.87E-112 | |  |
| 43 | LILRB4 | | 0.543407 | | 9.63E-147 | |  |
| 44 | APBB1IP | | 0.435081 | | 8.93E-89 | |  |
| 45 | GZMB | | 0.426183 | | 7.01E-85 | |  |
| 46 | PSMB9 | | 0.485846 | | 2.35E-113 | |  |
| 47 | GBP4 | | 0.500164 | | 4.52E-121 | |  |
| 48 | SOCS1 | | 0.44087 | | 2.26E-91 | |  |
| 49 | IFIT1 | | 0.431777 | | 2.58E-87 | |  |
| 50 | IFI30 | | 0.574699 | | 8.46E-168 | |  |
| 51 | PILRA | | 0.46936 | | 6.51E-105 | |  |
| 52 | CARD16 | | 0.440514 | | 3.27E-91 | |  |
| 53 | CD48 | | 0.41402 | | 9.64E-80 | |  |
| 54 | ICOS | | 0.474533 | | 1.64E-107 | |  |
| 55 | HLA-G | | 0.427915 | | 1.25E-85 | |  |
| 56 | GPR84 | | 0.486985 | | 5.91E-114 | |  |
| 57 | ISG15 | | 0.48152 | | 4.28E-111 | |  |
| 58 | GBP1 | | 0.546435 | | 1.10E-148 | |  |
| 59 | IL18BP | | 0.455232 | | 4.89E-98 | |  |
| 60 | PARP12 | | 0.414743 | | 4.83E-80 | |  |
| 61 | LPXN | | 0.422149 | | 3.75E-83 | |  |
| 62 | TYMP | | 0.40724 | | 5.72E-77 | |  |
| 63 | CMPK2 | | 0.446086 | | 9.34E-94 | |  |
| 64 | TYROBP | | 0.426745 | | 4.01E-85 | |  |
| 65 | LILRB3 | | 0.448332 | | 8.53E-95 | |  |
| 66 | ADAMDEC1 | | 0.508987 | | 5.18E-126 | |  |
| 67 | KLHL6 | | 0.401428 | | 1.21E-74 | |  |
| 68 | STAT1 | | 0.569722 | | 2.68E-164 | |  |
| 69 | IFI44 | | 0.552147 | | 2.12E-152 | |  |
| 70 | IFIT2 | | 0.527182 | | 1.13E-136 | |  |
| 71 | CD163 | | 0.446543 | | 5.75E-94 | |  |
| 72 | LAG3 | | 0.484929 | | 7.13E-113 | |  |
| 73 | FCGR1B | | 0.41868 | | 1.10E-81 | |  |
| 74 | GNGT2 | | 0.445622 | | 1.53E-93 | |  |
| 75 | CD38 | | 0.429954 | | 1.62E-86 | |  |
| 76 | C1QA | | 0.476026 | | 2.86E-108 | |  |
| 77 | P2RY6 | | 0.490552 | | 7.52E-116 | |  |
| 78 | CCL4 | | 0.441532 | | 1.13E-91 | |  |
| 79 | HK3 | | 0.473379 | | 6.28E-107 | |  |
| 80 | NCF1C | | 0.498948 | | 2.11E-120 | |  |
| 81 | LAP3 | | 0.435524 | | 5.68E-89 | |  |
| 82 | RSAD2 | | 0.557479 | | 6.20E-156 | |  |
| 83 | TNFAIP3 | | 0.429755 | | 1.98E-86 | |  |
| 84 | BU935198 | | 0.40314 | | 2.53E-75 | |  |
| 85 | MYO1G | | 0.44683 | | 4.24E-94 | |  |
| 86 | PLA2G7 | | 0.456374 | | 1.40E-98 | |  |
| 87 | IL32 | | 0.431295 | | 4.19E-87 | |  |
| 88 | CTSC | | 0.436307 | | 2.54E-89 | |  |
| 89 | PTPN7 | | 0.413222 | | 2.06E-79 | |  |
| 90 | ZNFX1 | | 0.402673 | | 3.89E-75 | |  |
| 91 | APOC1 | | 0.405276 | | 3.54E-76 | |  |
| 92 | ABI3 | | 0.444969 | | 3.05E-93 | |  |
| 93 | ARHGAP25 | | 0.433645 | | 3.87E-88 | |  |
| 94 | IGSF6 | | 0.454559 | | 1.02E-97 | |  |
| 95 | FCER1G | | 0.48057 | | 1.33E-110 | |  |
| 96 | IFI44L | | 0.547055 | | 4.39E-149 | |  |
| 97 | SOD2 | | 0.464658 | | 1.37E-102 | |  |
| 98 | ITGB2 | | 0.461715 | | 3.75E-101 | |  |
| 99 | ZBED2 | | 0.400159 | | 3.85E-74 | |  |
| 100 | LILRA5 | | 0.501314 | | 1.05E-121 | |  |
| 101 | NCF1 | | 0.439112 | | 1.41E-90 | |  |
| 102 | PTPRO | | 0.476246 | | 2.21E-108 | |  |
| 103 | IFI27 | | 0.474965 | | 9.89E-108 | |  |
| 104 | ITGB7 | | 0.439212 | | 1.27E-90 | |  |
| 105 | ADAP2 | | 0.429533 | | 2.47E-86 | |  |
| 106 | PLEKHO2 | | 0.415303 | | 2.83E-80 | |  |
| 107 | CD84 | | 0.425639 | | 1.20E-84 | |  |
| 108 | CTSL1 | | 0.428441 | | 7.39E-86 | |  |
| 109 | RIPK2 | | 0.400456 | | 2.94E-74 | |  |
| 110 | PARP14 | | 0.503752 | | 4.61E-123 | |  |
| 111 | CD72 | | 0.429508 | | 2.54E-86 | |  |
| 112 | SLC1A3 | | 0.41005 | | 4.13E-78 | |  |
| 113 | CD86 | | 0.594633 | | 1.91E-182 | |  |
| 114 | CD2 | | 0.416505 | | 8.94E-81 | |  |
| 115 | SLC15A3 | | 0.511803 | | 1.28E-127 | |  |
| 116 | CTSZ | | 0.453665 | | 2.70E-97 | |  |
| 117 | ST8SIA4 | | 0.426225 | | 6.73E-85 | |  |
| 118 | TRPV2 | | 0.465945 | | 3.20E-103 | |  |
| 119 | ARHGAP30 | | 0.439347 | | 1.10E-90 | |  |
| 120 | IL10RA | | 0.402808 | | 3.43E-75 | |  |
| 121 | ITGAX | | 0.408979 | | 1.13E-77 | |  |
| 122 | MYD88 | | 0.423533 | | 9.62E-84 | |  |
| 123 | IFIH1 | | 0.558443 | | 1.40E-156 | |  |
| 124 | TMEM140 | | 0.405393 | | 3.18E-76 | |  |
| 125 | SASH3 | | 0.428138 | | 1.00E-85 | |  |
| 126 | BATF2 | | 0.477376 | | 5.84E-109 | |  |
| 127 | CD300A | | 0.479009 | | 8.49E-110 | |  |
| 128 | PLEK | | 0.498316 | | 4.70E-120 | |  |
| 129 | SLC7A7 | | 0.562959 | | 1.24E-159 | |  |
| 130 | WARS | | 0.443626 | | 1.26E-92 | |  |
| 131 | MAFF | | 0.416299 | | 1.09E-80 | |  |
| 132 | SEMA4D | | 0.416507 | | 8.92E-81 | |  |
| 133 | TNFSF13B | | 0.578366 | | 2.04E-170 | |  |
| 134 | HLA-DMB | | 0.419659 | | 4.25E-82 | |  |
| 135 | CD83 | | 0.441209 | | 1.59E-91 | |  |
| 136 | MAFA | | 0.406949 | | 7.50E-77 | |  |
| 137 | HLA-F | | 0.474917 | | 1.05E-107 | |  |
| 138 | PIK3AP1 | | 0.400903 | | 1.96E-74 | |  |
| 139 | SIGLEC1 | | 0.411958 | | 6.82E-79 | |  |
| 140 | HLA-B | | 0.470538 | | 1.68E-105 | |  |
| 141 | FPR3 | | 0.579213 | | 5.01E-171 | |  |
| 142 | GNLY | | 0.422704 | | 2.17E-83 | |  |
| 143 | LAPTM5 | | 0.485573 | | 3.28E-113 | |  |
| 144 | CORO1A | | 0.449422 | | 2.65E-95 | |  |
| 145 | CD80 | | 1 | | 0 | |  |
| 146 | MCOLN2 | | 0.442562 | | 3.85E-92 | |  |
| 147 | MX2 | | 0.496471 | | 4.80E-119 | |  |
| 148 | CXCL9 | | 0.47879 | | 1.10E-109 | |  |
| 149 | TNFRSF9 | | 0.403875 | | 1.29E-75 | |  |
| 150 | DAPK1 | | 0.412998 | | 2.55E-79 | |  |
| 151 | EPSTI1 | | 0.631 | | 5.59E-212 | |  |
| 152 | PARP9 | | 0.426129 | | 7.40E-85 | |  |
| 153 | SPI1 | | 0.500069 | | 5.10E-121 | |  |
| 154 | TDO2 | | 0.424567 | | 3.47E-84 | |  |
| 155 | SLA | | 0.528445 | | 1.95E-137 | |  |
| 156 | C17orf87 | | 0.455002 | | 6.28E-98 | |  |
| 157 | C1QB | | 0.501016 | | 1.53E-121 | |  |
| 158 | FERMT3 | | 0.510668 | | 5.70E-127 | |  |
| 159 | NLRC5 | | 0.446466 | | 6.24E-94 | |  |
| 160 | LAIR2 | | 0.511375 | | 2.25E-127 | |  |
| 161 | IRF1 | | 0.42384 | | 7.11E-84 | |  |
| 162 | CXCL10 | | 0.614866 | | 2.13E-198 | |  |
| 163 | ISG20 | | 0.433323 | | 5.37E-88 | |  |
| 164 | KBTBD8 | | 0.431403 | | 3.76E-87 | |  |
| 165 | ETV7 | | 0.422221 | | 3.49E-83 | |  |
| 166 | BTK | | 0.413708 | | 1.30E-79 | |  |
| 167 | CTLA4 | | 0.495041 | | 2.87E-118 | |  |
| 168 | SP140 | | 0.519457 | | 4.57E-132 | |  |
| 169 | GPSM3 | | 0.407152 | | 6.20E-77 | |  |
| 170 | NCF4 | | 0.455694 | | 2.94E-98 | |  |
| 171 | CXCR6 | | 0.427939 | | 1.22E-85 | |  |
| 172 | TRIM21 | | 0.45554 | | 3.49E-98 | |  |
| 173 | CCL8 | | 0.521267 | | 3.90E-133 | |  |
| 174 | BG205162 | | 0.452035 | | 1.59E-96 | |  |
| 175 | CD4 | | 0.457117 | | 6.17E-99 | |  |
| 176 | EVI2B | | 0.406017 | | 1.78E-76 | |  |
| 177 | CASP1 | | 0.411522 | | 1.03E-78 | |  |
| 178 | C1orf162 | | 0.420942 | | 1.22E-82 | |  |
| 179 | FGD2 | | 0.430486 | | 9.48E-87 | |  |
| 180 | CCR1 | | 0.526201 | | 4.42E-136 | |  |
| 181 | LAMP3 | | 0.468823 | | 1.20E-104 | |  |
| 182 | LYN | | 0.520487 | | 1.13E-132 | |  |
| 183 | TAP1 | | 0.546292 | | 1.36E-148 | |  |
| 184 | SLC2A5 | | 0.456681 | | 9.97E-99 | |  |
| 185 | GPR65 | | 0.423341 | | 1.16E-83 | |  |
| 186 | CCL5 | | 0.40198 | | 7.33E-75 | |  |
| 187 | NFS1 | | 0.413559 | | 1.49E-79 | |  |
| 188 | DDX60 | | 0.401137 | | 1.58E-74 | |  |
| 189 | OASL | | 0.554998 | | 2.78E-154 | |  |
| 190 | NCKAP1L | | 0.456602 | | 1.09E-98 | |  |

| **Table S3 The immune-related positively correlated genes lists** | | | | | |
| --- | --- | --- | --- | --- | --- |
| TCGA positive correlated genes | | |  | METABRIC postively correlated genes | |
| rho9 | ABCC4 | 0.407317 | rho13 | IL21R | 0.427221 |
| rho29 | ACSL4 | 0.491577 | rho15 | DOCK2 | 0.41785 |
| rho52 | ADAMDEC1 | 0.674545 | rho60 | WAS | 0.407612 |
| rho99 | AIF1 | 0.516284 | rho84 | IL2RA | 0.436725 |
| rho190 | APOC1 | 0.440211 | rho108 | OSCAR | 0.439025 |
| rho195 | APOL2 | 0.402382 | rho123 | GCH1 | 0.437572 |
| rho262 | B2M | 0.645578 | rho178 | IDO1 | 0.498673 |
| rho297 | BCL2A1 | 0.649133 | rho193 | XAF1 | 0.455507 |
| rho329 | BIRC3 | 0.517875 | rho204 | CD53 | 0.521284 |
| rho379 | BTK | 0.670569 | rho247 | NUP62 | 0.554027 |
| rho380 | BTLA | 0.496103 | rho299 | IFNG | 0.404669 |
| rho387 | C1QA | 0.537077 | rho325 | C2 | 0.479168 |
| rho388 | C1QB | 0.6229 | rho338 | APOE | 0.402685 |
| rho390 | C1QC | 0.63575 | rho388 | PSME2 | 0.447239 |
| rho407 | C1S | 0.438749 | rho397 | CYBB | 0.456222 |
| rho408 | C2 | 0.603373 | rho465 | IFI6 | 0.414974 |
| rho416 | C3AR1 | 0.688829 | rho498 | LTA | 0.405253 |
| rho423 | C5AR1 | 0.624728 | rho499 | HLA-E | 0.428656 |
| rho455 | CARD11 | 0.553948 | rho510 | HAMP | 0.420703 |
| rho457 | CARD16 | 0.464367 | rho695 | C1QC | 0.532259 |
| rho458 | CARD17 | 0.554811 | rho699 | RUNX3 | 0.402757 |
| rho464 | CASP1 | 0.529566 | rho732 | IFIT3 | 0.485537 |
| rho465 | CASP10 | 0.424351 | rho741 | FLVCR2 | 0.504183 |
| rho470 | CASP5 | 0.617408 | rho797 | MX1 | 0.483048 |
| rho477 | CASS4 | 0.521004 | rho859 | LILRB4 | 0.543407 |
| rho494 | CCL11 | 0.437867 | rho885 | GZMB | 0.426183 |
| rho495 | CCL13 | 0.4778 | rho897 | PSMB9 | 0.485846 |
| rho498 | CCL2 | 0.521903 | rho934 | SOCS1 | 0.44087 |
| rho507 | CCL3 | 0.450356 | rho951 | IFIT1 | 0.431777 |
| rho508 | CCL7 | 0.539744 | rho955 | IFI30 | 0.574699 |
| rho509 | CCL8 | 0.69244 | rho962 | CARD16 | 0.440514 |
| rho518 | CCR1 | 0.793837 | rho971 | CD48 | 0.41402 |
| rho520 | CCR2 | 0.499137 | rho986 | ICOS | 0.474533 |
| rho522 | CCR4 | 0.561078 | rho987 | HLA-G | 0.427915 |
| rho523 | CCR5 | 0.680788 | rho1051 | GBP1 | 0.546435 |
| rho525 | CCR7 | 0.409573 | rho1060 | IL18BP | 0.455232 |
| rho526 | CCR8 | 0.709617 | rho1118 | LPXN | 0.422149 |
| rho528 | CCRL2 | 0.580241 | rho1125 | TYMP | 0.40724 |
| rho529 | CD101 | 0.415163 | rho1154 | TYROBP | 0.426745 |
| rho534 | CD163 | 0.649763 | rho1175 | LILRB3 | 0.448332 |
| rho539 | CD180 | 0.664407 | rho1179 | ADAMDEC1 | 0.508987 |
| rho546 | CD2 | 0.555982 | rho1183 | KLHL6 | 0.401428 |
| rho548 | CD200R1 | 0.509826 | rho1240 | STAT1 | 0.569722 |
| rho553 | CD226 | 0.625808 | rho1248 | IFI44 | 0.552147 |
| rho554 | CD244 | 0.455944 | rho1269 | IFIT2 | 0.527182 |
| rho555 | CD247 | 0.478089 | rho1314 | CD163 | 0.446543 |
| rho557 | CD27 | 0.43351 | rho1317 | LAG3 | 0.484929 |
| rho558 | CD274 | 0.654401 | rho1344 | FCGR1B | 0.41868 |
| rho560 | CD28 | 0.582154 | rho1373 | CD38 | 0.429954 |
| rho563 | CD300A | 0.600909 | rho1431 | C1QA | 0.476026 |
| rho564 | CD300C | 0.59981 | rho1577 | TNFAIP3 | 0.429755 |
| rho565 | CD300E | 0.61663 | rho1595 | PLA2G7 | 0.456374 |
| rho568 | CD300LF | 0.646538 | rho1633 | IL32 | 0.431295 |
| rho572 | CD33 | 0.549247 | rho1641 | CTSC | 0.436307 |
| rho575 | CD37 | 0.541042 | rho1756 | APOC1 | 0.405276 |
| rho576 | CD38 | 0.58387 | rho1776 | IGSF6 | 0.454559 |
| rho577 | CD3D | 0.48429 | rho1781 | FCER1G | 0.48057 |
| rho578 | CD3E | 0.484137 | rho1794 | IFI44L | 0.547055 |
| rho580 | CD3G | 0.546596 | rho1813 | SOD2 | 0.464658 |
| rho581 | CD4 | 0.678562 | rho1814 | ITGB2 | 0.461715 |
| rho582 | CD40 | 0.459772 | rho1840 | LILRA5 | 0.501314 |
| rho583 | CD40LG | 0.410021 | rho1846 | NCF1 | 0.439112 |
| rho587 | CD48 | 0.561322 | rho1854 | IFI27 | 0.474965 |
| rho588 | CD5 | 0.518148 | rho1856 | ITGB7 | 0.439212 |
| rho589 | CD52 | 0.448512 | rho1918 | CD84 | 0.425639 |
| rho590 | CD53 | 0.722942 | rho1953 | CTSL1 | 0.428441 |
| rho595 | CD6 | 0.49433 | rho1961 | RIPK2 | 0.400456 |
| rho598 | CD69 | 0.444357 | rho1986 | CD72 | 0.429508 |
| rho599 | CD7 | 0.461523 | rho1993 | SLC1A3 | 0.41005 |
| rho600 | CD72 | 0.598852 | rho1995 | CD86 | 0.594633 |
| rho601 | CD74 | 0.537393 | rho2000 | CD2 | 0.416505 |
| rho604 | CD80 | 1 | rho2010 | CTSZ | 0.453665 |
| rho607 | CD83 | 0.641361 | rho2120 | IL10RA | 0.402808 |
| rho608 | CD84 | 0.722943 | rho2124 | ITGAX | 0.408979 |
| rho609 | CD86 | 0.817542 | rho2151 | MYD88 | 0.423533 |
| rho610 | CD8A | 0.452624 | rho2163 | IFIH1 | 0.558443 |
| rho611 | CD8B | 0.401831 | rho2272 | CD300A | 0.479009 |
| rho614 | CD96 | 0.555731 | rho2281 | PLEK | 0.498316 |
| rho688 | CERKL | 0.444507 | rho2336 | SEMA4D | 0.416507 |
| rho721 | CHST11 | 0.642594 | rho2363 | TNFSF13B | 0.578366 |
| rho732 | CIITA | 0.537839 | rho2370 | HLA-DMB | 0.419659 |
| rho763 | CLEC4A | 0.576504 | rho2393 | CD83 | 0.441209 |
| rho767 | CLEC7A | 0.740121 | rho2459 | HLA-F | 0.474917 |
| rho772 | CLNK | 0.469341 | rho2497 | SIGLEC1 | 0.411958 |
| rho784 | CMKLR1 | 0.476801 | rho2511 | HLA-B | 0.470538 |
| rho837 | COL6A5 | 0.481741 | rho2515 | FPR3 | 0.579213 |
| rho849 | CORO1A | 0.464422 | rho2531 | GNLY | 0.422704 |
| rho850 | CORO1C | 0.499202 | rho2553 | CORO1A | 0.449422 |
| rho851 | COTL1 | 0.406204 | rho2556 | CD80 | 1 |
| rho860 | CR1 | 0.446495 | rho2626 | CXCL9 | 0.47879 |
| rho861 | CR1L | 0.56034 | rho2648 | TNFRSF9 | 0.403875 |
| rho874 | CRTAM | 0.567999 | rho2670 | DAPK1 | 0.412998 |
| rho879 | CSF1R | 0.544646 | rho2707 | SPI1 | 0.500069 |
| rho881 | CSF2RA | 0.512596 | rho2789 | C1QB | 0.501016 |
| rho882 | CSF2RB | 0.516498 | rho2845 | FERMT3 | 0.510668 |
| rho895 | CTLA4 | 0.675764 | rho2857 | NLRC5 | 0.446466 |
| rho906 | CTSB | 0.48471 | rho2901 | LAIR2 | 0.511375 |
| rho907 | CTSC | 0.54139 | rho2925 | IRF1 | 0.42384 |
| rho915 | CTSS | 0.668488 | rho2946 | CXCL10 | 0.614866 |
| rho916 | CTSW | 0.401328 | rho2992 | ISG20 | 0.433323 |
| rho930 | CXCL10 | 0.74004 | rho3030 | BTK | 0.413708 |
| rho931 | CXCL11 | 0.697867 | rho3045 | CTLA4 | 0.495041 |
| rho933 | CXCL13 | 0.418406 | rho3145 | CXCR6 | 0.427939 |
| rho941 | CXCL9 | 0.563522 | rho3201 | CCL8 | 0.521267 |
| rho944 | CXCR3 | 0.503486 | rho3262 | CD4 | 0.457117 |
| rho947 | CXCR6 | 0.59485 | rho3385 | CASP1 | 0.411522 |
| rho949 | CYBB | 0.730124 | rho3463 | FGD2 | 0.430486 |
| rho961 | CYTIP | 0.531013 | rho3473 | CCR1 | 0.526201 |
| rho969 | DAPK1 | 0.531769 | rho3494 | TAP1 | 0.546292 |
| rho996 | DDX58 | 0.497883 | rho3579 | GPR65 | 0.423341 |
| rho1053 | DGKG | 0.42747 |  |  |  |
| rho1066 | DLEU7 | 0.588897 |  |  |  |
| rho1089 | DOCK10 | 0.476447 |  |  |  |
| rho1090 | DOCK11 | 0.499194 |  |  |  |
| rho1091 | DOCK2 | 0.69526 |  |  |  |
| rho1097 | DOCK8 | 0.492233 |  |  |  |
| rho1104 | DRAM1 | 0.425376 |  |  |  |
| rho1157 | EGFL6 | 0.446936 |  |  |  |
| rho1166 | EIF2AK2 | 0.491033 |  |  |  |
| rho1182 | EMILIN2 | 0.575236 |  |  |  |
| rho1189 | EOMES | 0.475412 |  |  |  |
| rho1245 | F5 | 0.546483 |  |  |  |
| rho1268 | FAS | 0.424571 |  |  |  |
| rho1269 | FASLG | 0.557017 |  |  |  |
| rho1284 | FCAR | 0.518857 |  |  |  |
| rho1286 | FCER1G | 0.686521 |  |  |  |
| rho1289 | FCGR1A | 0.679503 |  |  |  |
| rho1290 | FCGR1B | 0.618536 |  |  |  |
| rho1291 | FCGR2A | 0.626885 |  |  |  |
| rho1292 | FCGR2B | 0.485457 |  |  |  |
| rho1293 | FCGR3A | 0.740713 |  |  |  |
| rho1294 | FCGR3B | 0.434052 |  |  |  |
| rho1305 | FCRL3 | 0.478564 |  |  |  |
| rho1307 | FCRL5 | 0.410816 |  |  |  |
| rho1315 | FERMT3 | 0.594719 |  |  |  |
| rho1322 | FGD2 | 0.625896 |  |  |  |
| rho1360 | FLVCR2 | 0.642108 |  |  |  |
| rho1390 | FOXP3 | 0.618776 |  |  |  |
| rho1393 | FPR1 | 0.567588 |  |  |  |
| rho1394 | FPR2 | 0.673075 |  |  |  |
| rho1395 | FPR3 | 0.791827 |  |  |  |
| rho1413 | FUT4 | 0.432168 |  |  |  |
| rho1439 | GBP1 | 0.70333 |  |  |  |
| rho1443 | GCH1 | 0.434989 |  |  |  |
| rho1462 | GIMAP5 | 0.418108 |  |  |  |
| rho1483 | GNLY | 0.482781 |  |  |  |
| rho1494 | GPNMB | 0.509593 |  |  |  |
| rho1496 | GPR183 | 0.520904 |  |  |  |
| rho1497 | GPR65 | 0.70306 |  |  |  |
| rho1519 | GZMA | 0.473187 |  |  |  |
| rho1520 | GZMB | 0.558675 |  |  |  |
| rho1521 | GZMH | 0.400854 |  |  |  |
| rho1522 | GZMK | 0.412716 |  |  |  |
| rho1525 | HAMP | 0.405838 |  |  |  |
| rho1534 | HAVCR2 | 0.770855 |  |  |  |
| rho1542 | HCK | 0.635404 |  |  |  |
| rho1570 | HIVEP2 | 0.406989 |  |  |  |
| rho1572 | HLA-A | 0.446559 |  |  |  |
| rho1573 | HLA-B | 0.518077 |  |  |  |
| rho1575 | HLA-DMA | 0.515832 |  |  |  |
| rho1576 | HLA-DMB | 0.63919 |  |  |  |
| rho1577 | HLA-DOA | 0.528006 |  |  |  |
| rho1579 | HLA-DPA1 | 0.526487 |  |  |  |
| rho1580 | HLA-DPB1 | 0.434952 |  |  |  |
| rho1581 | HLA-DQA1 | 0.597875 |  |  |  |
| rho1583 | HLA-DQB1 | 0.449502 |  |  |  |
| rho1585 | HLA-DRA | 0.598422 |  |  |  |
| rho1586 | HLA-DRB1 | 0.522712 |  |  |  |
| rho1587 | HLA-DRB5 | 0.458817 |  |  |  |
| rho1588 | HLA-E | 0.44625 |  |  |  |
| rho1589 | HLA-F | 0.45703 |  |  |  |
| rho1596 | HMOX1 | 0.47967 |  |  |  |
| rho1644 | ICAM1 | 0.516561 |  |  |  |
| rho1646 | ICAM3 | 0.471811 |  |  |  |
| rho1650 | ICOS | 0.710425 |  |  |  |
| rho1656 | IDO1 | 0.614908 |  |  |  |
| rho1658 | IFI16 | 0.539997 |  |  |  |
| rho1659 | IFI27 | 0.499773 |  |  |  |
| rho1662 | IFI30 | 0.553588 |  |  |  |
| rho1664 | IFI44 | 0.633954 |  |  |  |
| rho1665 | IFI44L | 0.661836 |  |  |  |
| rho1666 | IFI6 | 0.429947 |  |  |  |
| rho1667 | IFIH1 | 0.640293 |  |  |  |
| rho1668 | IFIT1 | 0.490679 |  |  |  |
| rho1670 | IFIT2 | 0.631452 |  |  |  |
| rho1671 | IFIT3 | 0.673978 |  |  |  |
| rho1672 | IFIT5 | 0.443007 |  |  |  |
| rho1691 | IFNAR2 | 0.444183 |  |  |  |
| rho1694 | IFNG | 0.555902 |  |  |  |
| rho1721 | IGF2BP3 | 0.514805 |  |  |  |
| rho1736 | IGSF6 | 0.634034 |  |  |  |
| rho1746 | IKZF1 | 0.618526 |  |  |  |
| rho1747 | IL10 | 0.711394 |  |  |  |
| rho1748 | IL10RA | 0.635061 |  |  |  |
| rho1753 | IL12B | 0.437385 |  |  |  |
| rho1754 | IL12RB1 | 0.648512 |  |  |  |
| rho1755 | IL12RB2 | 0.463928 |  |  |  |
| rho1759 | IL15 | 0.5163 |  |  |  |
| rho1760 | IL15RA | 0.523504 |  |  |  |
| rho1761 | IL16 | 0.445646 |  |  |  |
| rho1773 | IL18 | 0.424363 |  |  |  |
| rho1774 | IL18BP | 0.553718 |  |  |  |
| rho1775 | IL18R1 | 0.436349 |  |  |  |
| rho1776 | IL18RAP | 0.500355 |  |  |  |
| rho1778 | IL1A | 0.466268 |  |  |  |
| rho1779 | IL1B | 0.503898 |  |  |  |
| rho1793 | IL21 | 0.447045 |  |  |  |
| rho1794 | IL21R | 0.666021 |  |  |  |
| rho1805 | IL2RA | 0.704828 |  |  |  |
| rho1806 | IL2RB | 0.566757 |  |  |  |
| rho1807 | IL2RG | 0.578815 |  |  |  |
| rho1811 | IL32 | 0.438825 |  |  |  |
| rho1821 | IL4I1 | 0.65333 |  |  |  |
| rho1828 | IL7 | 0.433406 |  |  |  |
| rho1829 | IL7R | 0.496588 |  |  |  |
| rho1831 | IL9R | 0.458761 |  |  |  |
| rho1847 | INPP5D | 0.481235 |  |  |  |
| rho1858 | IRF1 | 0.517229 |  |  |  |
| rho1863 | IRF4 | 0.507766 |  |  |  |
| rho1864 | IRF5 | 0.406675 |  |  |  |
| rho1867 | IRF8 | 0.599819 |  |  |  |
| rho1887 | ITGA4 | 0.579974 |  |  |  |
| rho1895 | ITGAL | 0.460886 |  |  |  |
| rho1896 | ITGAM | 0.545222 |  |  |  |
| rho1898 | ITGAX | 0.658962 |  |  |  |
| rho1902 | ITGB2 | 0.628513 |  |  |  |
| rho1908 | ITGB7 | 0.592708 |  |  |  |
| rho1913 | ITK | 0.554435 |  |  |  |
| rho1926 | JAK3 | 0.473228 |  |  |  |
| rho1928 | JAKMIP2 | 0.460842 |  |  |  |
| rho1956 | KIR2DL4 | 0.53739 |  |  |  |
| rho1958 | KIR3DL2 | 0.431257 |  |  |  |
| rho1971 | KLHL6 | 0.667499 |  |  |  |
| rho1985 | KYNU | 0.48756 |  |  |  |
| rho1988 | LAG3 | 0.594683 |  |  |  |
| rho1989 | LAIR1 | 0.705026 |  |  |  |
| rho1990 | LAIR2 | 0.517964 |  |  |  |
| rho2006 | LAT2 | 0.533831 |  |  |  |
| rho2008 | LAX1 | 0.541522 |  |  |  |
| rho2010 | LCK | 0.486588 |  |  |  |
| rho2012 | LCP1 | 0.498029 |  |  |  |
| rho2013 | LCP2 | 0.743427 |  |  |  |
| rho2040 | LILRA4 | 0.426242 |  |  |  |
| rho2041 | LILRA5 | 0.662462 |  |  |  |
| rho2042 | LILRA6 | 0.681954 |  |  |  |
| rho2043 | LILRB1 | 0.751076 |  |  |  |
| rho2044 | LILRB2 | 0.72467 |  |  |  |
| rho2045 | LILRB3 | 0.637547 |  |  |  |
| rho2046 | LILRB4 | 0.725727 |  |  |  |
| rho2047 | LILRB5 | 0.40591 |  |  |  |
| rho2048 | LIMS1 | 0.442963 |  |  |  |
| rho2066 | LPXN | 0.631185 |  |  |  |
| rho2075 | LRMP | 0.412951 |  |  |  |
| rho2098 | LST1 | 0.463725 |  |  |  |
| rho2099 | LTA | 0.604394 |  |  |  |
| rho2121 | LY86 | 0.481973 |  |  |  |
| rho2122 | LY9 | 0.461748 |  |  |  |
| rho2123 | LY96 | 0.491432 |  |  |  |
| rho2156 | MAP4K1 | 0.460692 |  |  |  |
| rho2204 | MERTK | 0.515599 |  |  |  |
| rho2240 | MNDA | 0.720435 |  |  |  |
| rho2246 | MPEG1 | 0.607684 |  |  |  |
| rho2249 | MPP1 | 0.546234 |  |  |  |
| rho2265 | MSN | 0.459339 |  |  |  |
| rho2266 | MSR1 | 0.67982 |  |  |  |
| rho2282 | MX1 | 0.558937 |  |  |  |
| rho2290 | MYD88 | 0.44062 |  |  |  |
| rho2313 | NCF1 | 0.610306 |  |  |  |
| rho2314 | NCF2 | 0.715055 |  |  |  |
| rho2315 | NCK1 | 0.433847 |  |  |  |
| rho2319 | NCR1 | 0.457695 |  |  |  |
| rho2321 | NCR3 | 0.408603 |  |  |  |
| rho2347 | NFAM1 | 0.516261 |  |  |  |
| rho2381 | NKG7 | 0.452644 |  |  |  |
| rho2395 | NLRC3 | 0.416045 |  |  |  |
| rho2396 | NLRC4 | 0.652892 |  |  |  |
| rho2397 | NLRC5 | 0.657514 |  |  |  |
| rho2401 | NLRP3 | 0.443404 |  |  |  |
| rho2444 | NRP2 | 0.425972 |  |  |  |
| rho2450 | NT5E | 0.474513 |  |  |  |
| rho2468 | OLR1 | 0.644291 |  |  |  |
| rho2480 | OSCAR | 0.602854 |  |  |  |
| rho2481 | OSM | 0.504752 |  |  |  |
| rho2490 | P2RX7 | 0.590801 |  |  |  |
| rho2502 | PAG1 | 0.569254 |  |  |  |
| rho2510 | PARVG | 0.51561 |  |  |  |
| rho2511 | PATL1 | 0.402515 |  |  |  |
| rho2591 | PDCD1 | 0.478722 |  |  |  |
| rho2594 | PDCD1LG2 | 0.743223 |  |  |  |
| rho2649 | PIK3CG | 0.623192 |  |  |  |
| rho2653 | PIM2 | 0.402478 |  |  |  |
| rho2677 | PLA2G7 | 0.569915 |  |  |  |
| rho2682 | PLAU | 0.420826 |  |  |  |
| rho2683 | PLAUR | 0.487559 |  |  |  |
| rho2689 | PLEK | 0.742064 |  |  |  |
| rho2699 | PLSCR1 | 0.594801 |  |  |  |
| rho2701 | PLXNC1 | 0.481073 |  |  |  |
| rho2764 | PRF1 | 0.498631 |  |  |  |
| rho2783 | PRKCB | 0.509417 |  |  |  |
| rho2789 | PRKCQ | 0.48393 |  |  |  |
| rho2833 | PSMB9 | 0.549919 |  |  |  |
| rho2863 | PSTPIP1 | 0.477642 |  |  |  |
| rho2864 | PTAFR | 0.602167 |  |  |  |
| rho2866 | PTCRA | 0.414091 |  |  |  |
| rho2874 | PTGER4 | 0.451016 |  |  |  |
| rho2891 | PTPN22 | 0.708095 |  |  |  |
| rho2893 | PTPRC | 0.690867 |  |  |  |
| rho2981 | RELT | 0.435139 |  |  |  |
| rho3062 | RUNX3 | 0.488447 |  |  |  |
| rho3114 | SELL | 0.47325 |  |  |  |
| rho3116 | SELPLG | 0.552725 |  |  |  |
| rho3127 | SEMA4D | 0.43137 |  |  |  |
| rho3132 | SEMA7A | 0.424668 |  |  |  |
| rho3145 | SERPINB9 | 0.592253 |  |  |  |
| rho3166 | SH2D1A | 0.528067 |  |  |  |
| rho3172 | SH3KBP1 | 0.403295 |  |  |  |
| rho3182 | SIGLEC1 | 0.750103 |  |  |  |
| rho3183 | SIGLEC10 | 0.664351 |  |  |  |
| rho3186 | SIGLEC14 | 0.592742 |  |  |  |
| rho3187 | SIGLEC5 | 0.516057 |  |  |  |
| rho3189 | SIGLEC7 | 0.710086 |  |  |  |
| rho3191 | SIGLEC9 | 0.672246 |  |  |  |
| rho3196 | SIRPA | 0.473407 |  |  |  |
| rho3197 | SIRPG | 0.549292 |  |  |  |
| rho3199 | SIT1 | 0.46739 |  |  |  |
| rho3208 | SLA2 | 0.564786 |  |  |  |
| rho3209 | SLAMF1 | 0.527709 |  |  |  |
| rho3210 | SLAMF7 | 0.601165 |  |  |  |
| rho3212 | SLC11A1 | 0.438781 |  |  |  |
| rho3221 | SLC1A3 | 0.592323 |  |  |  |
| rho3236 | SLFN11 | 0.486511 |  |  |  |
| rho3238 | SLFN12L | 0.607188 |  |  |  |
| rho3241 | SLFN5 | 0.415563 |  |  |  |
| rho3284 | SOAT1 | 0.430133 |  |  |  |
| rho3293 | SOD2 | 0.489298 |  |  |  |
| rho3306 | SP100 | 0.503278 |  |  |  |
| rho3319 | SPI1 | 0.580201 |  |  |  |
| rho3323 | SPN | 0.582419 |  |  |  |
| rho3334 | SRGN | 0.641678 |  |  |  |
| rho3348 | STAT1 | 0.712137 |  |  |  |
| rho3349 | STAT2 | 0.573318 |  |  |  |
| rho3351 | STAT4 | 0.527693 |  |  |  |
| rho3358 | STK17A | 0.489126 |  |  |  |
| rho3359 | STK17B | 0.492239 |  |  |  |
| rho3363 | STK4 | 0.481498 |  |  |  |
| rho3398 | TAGAP | 0.644596 |  |  |  |
| rho3402 | TAP1 | 0.614575 |  |  |  |
| rho3403 | TAP2 | 0.566907 |  |  |  |
| rho3416 | TBX21 | 0.49376 |  |  |  |
| rho3446 | TGFBI | 0.463458 |  |  |  |
| rho3460 | THEMIS | 0.490078 |  |  |  |
| rho3461 | THEMIS2 | 0.611545 |  |  |  |
| rho3477 | TIGIT | 0.63074 |  |  |  |
| rho3478 | TIMD4 | 0.571741 |  |  |  |
| rho3490 | TLR1 | 0.625217 |  |  |  |
| rho3492 | TLR2 | 0.561737 |  |  |  |
| rho3494 | TLR4 | 0.56296 |  |  |  |
| rho3496 | TLR6 | 0.5134 |  |  |  |
| rho3497 | TLR7 | 0.604046 |  |  |  |
| rho3498 | TLR8 | 0.644319 |  |  |  |
| rho3518 | TNFAIP3 | 0.534728 |  |  |  |
| rho3520 | TNFAIP8 | 0.464045 |  |  |  |
| rho3522 | TNFAIP8L2 | 0.537724 |  |  |  |
| rho3538 | TNFRSF1B | 0.491839 |  |  |  |
| rho3543 | TNFRSF8 | 0.409406 |  |  |  |
| rho3544 | TNFRSF9 | 0.701176 |  |  |  |
| rho3550 | TNFSF13B | 0.778435 |  |  |  |
| rho3551 | TNFSF14 | 0.543065 |  |  |  |
| rho3554 | TNFSF4 | 0.466108 |  |  |  |
| rho3555 | TNFSF8 | 0.670261 |  |  |  |
| rho3556 | TNFSF9 | 0.421777 |  |  |  |
| rho3560 | TNIP3 | 0.584725 |  |  |  |
| rho3575 | TOX | 0.41296 |  |  |  |
| rho3591 | TRAF1 | 0.405472 |  |  |  |
| rho3603 | TRAT1 | 0.504458 |  |  |  |
| rho3604 | TREM1 | 0.445583 |  |  |  |
| rho3607 | TREML2 | 0.472123 |  |  |  |
| rho3617 | TRIM69 | 0.554627 |  |  |  |
| rho3651 | TYROBP | 0.516731 |  |  |  |
| rho3653 | UBASH3A | 0.49514 |  |  |  |
| rho3654 | UBD | 0.462647 |  |  |  |
| rho3699 | VAV1 | 0.522304 |  |  |  |
| rho3702 | VCAM1 | 0.596751 |  |  |  |
| rho3714 | VNN1 | 0.43669 |  |  |  |
| rho3725 | VSIG4 | 0.436013 |  |  |  |
| rho3730 | WAS | 0.520503 |  |  |  |
| rho3744 | WNT2 | 0.424445 |  |  |  |
| rho3759 | XAF1 | 0.639039 |  |  |  |
| rho3760 | XCL1 | 0.448783 |  |  |  |
| rho3761 | XCL2 | 0.437228 |  |  |  |
| rho3765 | XIRP1 | 0.578644 |  |  |  |
| rho3788 | ZAP70 | 0.40967 |  |  |  |
| rho3789 | ZBP1 | 0.646027 |  |  |  |
| rho3798 | ZEB2 | 0.451454 |  |  |  |

| **Table S4 Seven clusters of 104 genes defined as metagenes.** | | | | | |  |
| --- | --- | --- | --- | --- | --- | --- |
| HCK | IgG | Interferon | LCK | MHC_I | MHC_II | STAT1 |
| C1QB | IGSF8 | IFIT1 | CD2 | HLA-E | HLA-DRB1 | TAP1 |
| C1QA | ISLR2 | IFIT3 | GZMK | HLA-H | HLA-DRB5 | STAT1 |
| AIF1 | IGSF21 | IFI44L | GZMA | HLA-B | HLA-DRB3 | CXCL10 |
| LST1 | IGSF1 | OAS3 | CD3D | HLA-J | HLA-DPA1 | CXCL11 |
| DOCK2 | IGSF22 | MX1 | CD53 | HLA-F | HLA-DRA | GBP1 |
| LAPTM5 | IGDCC3 | RSAD2 | LCK | HLA-G | HLA-DQA1 | CXCL9 |
| TYROBP | IGHD | IFI44 | ARHGAP15 | HLA-A | HLA-DQA2 | |
| MS4A4A | IGSF11 | OAS2 | CCL5 | HLA-C | HLA-DMA | |
| MS4A6A | IGSF5 | OAS1 | GMFG | HLA-L | HLA-DOA | |
| CD163 | IGSF6 |  | SELL |  | HLA-DRB4 | |
| ITGB2 |  |  | STAT4 |  | HLA-DMB | |
| SLC7A7 |  |  | SAMSN1 |  | HLA-DQB1 | |
| LAIR1 |  |  | RAC2 |  | HLA-DPB1 | |
| HCK |  |  | HCLS1 |  | HLA-DQB2 | |
| TFEC |  |  | CCR7 |  | CD74 |  |
| IFI30 |  |  | PIK3CD |  | PTPRC |  |
| MNDA |  |  | CORO1A |  | HLA-DOB | |
| FCER1G |  |  | CD48 |  | HLA-DPB2 | |
| RNASE6 |  |  | IL2RG |  |  |  |
| SLCO2B1 |  |  | SH2D1A |  |  |  |
| CCR1 |  |  | SLAMF1 |  |  |  |
|  |  |  | IL7R |  |  |  |
|  |  |  | INPP5D |  |  |  |
|  |  |  | KLRK1 |  |  |  |
|  |  |  | FGL2 |  |  |  |
|  |  |  | IRF8 |  |  |  |
|  |  |  | SELPLG |  |  |  |
|  |  |  | IL10RA |  |  |  |
|  |  |  | SLA |  |  |  |
|  |  |  | CCR2 |  |  |  |
|  |  |  | CSF2RB |  |  |  |
